# megaTALs: a rare-cleaving nuclease architecture for therapeutic genome engineering

**DOI:** 10.1093/nar/gkt1224

**Published:** 2013-11-26

**Authors:** Sandrine Boissel, Jordan Jarjour, Alexander Astrakhan, Andrew Adey, Agnès Gouble, Philippe Duchateau, Jay Shendure, Barry L. Stoddard, Michael T. Certo, David Baker, Andrew M. Scharenberg

**Affiliations:** ^1^Molecular and Cellular Biology Program, University of Washington, Seattle, WA 98195, USA, ^2^Center for Immunity and Immunotherapies, Seattle Children’s Research Institute, Seattle, WA 98101, USA, ^3^Pregenen, Inc., Seattle, WA 98103, USA, ^4^Department of Genome Sciences, University of Washington, Seattle, WA 98195, USA, ^5^Cellectis S.A., Paris, 75013, France, ^6^Division of Basic Sciences, Fred Hutch Cancer Research Center, Seattle, WA 98109, USA, ^7^Department of Biochemistry, University of Washington, Seattle, WA 98195, USA, ^8^Howard Hughes Medical Institute, University of Washington, Seattle, WA 98195, USA and ^9^Department of Immunology, University of Washington, Seattle, WA 98195, USA

## Abstract

Rare-cleaving endonucleases have emerged as important tools for making targeted genome modifications. While multiple platforms are now available to generate reagents for research applications, each existing platform has significant limitations in one or more of three key properties necessary for therapeutic application: efficiency of cleavage at the desired target site, specificity of cleavage (i.e. rate of cleavage at ‘off-target’ sites), and efficient/facile means for delivery to desired target cells. Here, we describe the development of a single-chain rare-cleaving nuclease architecture, which we designate ‘megaTAL’, in which the DNA binding region of a transcription activator-like (TAL) effector is used to ‘address’ a site-specific meganuclease adjacent to a single desired genomic target site. This architecture allows the generation of extremely active and hyper-specific compact nucleases that are compatible with all current viral and nonviral cell delivery methods.

## INTRODUCTION

Targeted genome modifications can be achieved using rare cleaving nucleases, such as zinc-finger nucleases (ZFNs), transcription activator-like effector nucleases (TALENs), meganucleases (mns, also termed homing endonucleases) and clustered, regularly interspaced, short palindromic repeat (CRISPR) RNA-guided nucleases (1–9). These reagents allow precise alterations to be introduced at their DNA recognition sites through cleavage of both DNA strands to yield double-strand breaks (DSBs) that are recognized and processed by the nonhomologous end joining (NHEJ) or homologous recombination (HR) DNA repair pathway (10–12). Repair by NHEJ involves religation of the DNA ends, which, after repeated cleavage, is often accompanied by mutagenesis via the introduction of small insertions or deletions (indels) at the site of the DSB that can result in the disruption of the coding sequence of a gene. In contrast, repair by HR results in seamless modification of the genome by copying an appropriate homologous DNA template at the site of the DNA break.

The most demanding application of rare-cleaving nucleases is their use for therapeutic genome editing. For these applications, nuclease reagents must be delivered to a desired target cell in sufficient quantity to efficiently generate the desired DNA modification, while maintaining exquisite specificity to avoid compromising the genomic integrity of the cell. While the latest advances in each of the above-mentioned nuclease platforms enable quality nuclease reagents that are suitable for nearly all research and nontherapeutic commercial applications to be generated for little cost (13–20), several considerations still remain that limit their use for human cell therapies. These include a significant potential for off-target cleavage, particularly if expressed at high levels, as has been observed in the therapeutic application of ZFNs for disruption of the CCR5 gene in human T-cells (21–23). Additionally, since the FokI cleavage domain used in ZFNs and TALENs is activated via dimerization, ZFN and TALEN technologies require the construction and delivery of two protein halves, each comprising a distinct DNA binding domain fused to FokI, for a single target site. This architectural requirement limits the potential for ZFN and TALEN delivery into primary cells using viral vectors as well as their simultaneous (i.e. multiplexed) delivery of two or more nucleases to modify more than one gene (24,25). Although CRISPR systems have shown exciting promise in developing reagents for genome engineering with easily designed target recognition, they exhibit high off-target cleavage when used with single-guide RNAs, thus necessitating the use of nicking enzymes and dual guide RNAs per target to achieve specificity equivalent to that of the other major platforms (26–28). The consequent need to deliver both the Cas9 ORF plus two guides per target would appear to pose significant limitations for the use of CRISPR in therapeutic applications requiring viral vectorization as well as for applications requiring multiplexing. Finally, although architecturally well suited for therapeutic applications, mns’ tightly coupled cleavage and binding activity have limited their potential for re-design toward novel DNA target sites, with the majority of redesign efforts resulting in respecified enzymes with low target site affinity and consequent low overall activity (29,30).

To address the limitations of present nuclease platforms, we have developed a hybrid nuclease architecture that combines the ease of engineerability of a TAL effector (TALE) with the cleavage sequence specificity of a mn cleavage domain. This ‘megaTAL’ architecture was achieved by fusing minimal TAL effector domains to the N-terminus of mns derived from the LAGLIDADG homing endonuclease family.

## MATERIALS AND METHODS

### megaTAL and mn construct generation

MegaTALs were constructed using the Golden Gate assembly strategy previously described by Cermak *et al.*, using an repeat variable diresidue (RVD) plasmid library and destination vector generously provided by the Voytas lab (17). Plasmids were modified to allow the assembly of TAL effectors 1.5–10.5 RVDs long. The pthX01 destination vector was modified to include a hemagglutinin (HA) tag immediately downstream of the nuclear localization signal (NLS) and to yield a NΔ154, C+63 TALEN scaffold. TAL effectors were built using the following RVDs to target each specific nucleotide: A—NI, C—HD, G—NN and T—NG. Following cloning of the TAL effector repeats into the destination vector, the protein linkers (Fok: SQLVKS, Zn4: VGGS, Flex1: GGSG or Flex2: GGSGGGSG) and I-AniI or I-OnuI mn variants were cloned in place of the FokI nuclease catalytic domain between the Xba-I and Sal-I restriction sites. All megaTALs, except those used to compare linker activity, were made using the Zn4 protein linker. The codon diverged 6.5C-WT megaTAL (Supplementary Figure S2d) was constructed by gene synthesizing a TAL effector repeat array (Invitrogen) that was then cloned between Age-I and Xho-I sites cloned into the Golden Gate destination vector by site-directed mutagenesis. Control constructs expressing mn variants were made by cloning the nuclease downstream of an NLS and HA tag between the Sbf-I and Sal-I restriction sites. All constructs, except those used for mRNA production, encode BFP-T2A-nuclease for tracking of nuclease expression during flow cytometry. An example of a full megaTAL protein sequence (L538-Zn4-Y2 I-AniI) as well as the sequence for each mn tested and the RVDs used for each synthetic TAL effector are provided in Supplementary Figure S6.

### Lentiviral production

Lentivirus (LV) and integration-deficient LV (IDLV) were generated as previously described (31). Briefly, 9 × 10^6^ cells were transfected in 10-cm dishes with 6 μg of the nuclease construct, 3 μg of psPAX2 viral packaging plasmid and 1.5 μg of either pMD2G (for LV) or integration-deficient D64V pMD2G (for IDLV) viral envelope plasmid using PEI (Polysciences). Two days after transfection, viral supernatants were collected and concentrated by centrifugation. Viral titers were determined from the population of blue fluorescent protein (BFP) expressing cells 72 h after transducing 293T cells with varying volumes of concentrated virus using polybrene.

### Cell line derivation

HEK293T cell lines were generated harboring either the traffic light reporter [TLR 2.1, (32)] or a modified traffic light reporter (TLR) containing an near-infrared fluorescent protein (iRFP) gene in place of puromycin [epigenetic TLR, (33)]. Cells were derived as previously described with slight modifications. Briefly, HEK293T cells were transduced with recombinant LV to yield 5–10% transduction, based on either iRFP expression (epigenetic TLR) or cell survival (TLR 2.1). Approximately 5 days after transduction, cells were sorted for iRFP+/mCherry− populations (epigenetic TLR) or mCherry− populations (TLR 2.1).

### Cell sorting and flow cytometry

Cells were analyzed by flow cytometry on the BD LSRII and sorted on the BD FACS ARIAII. Fluorophores were detected using the following lasers and filters: mCherry — excited 561 nm, acquired 610/20; mTagBFP—excited 405 nm, acquired 450/50; eGFP—excited 488 nm, acquired 525/50; iRFP—excited 640 nm, acquired 730/45. Data were analyzed using FlowJo software.

### TLR assay

The TLR assay was performed as previously described with slight modifications. Cells harboring the TLR or epigenetic TLR 2.1 were plated at 1–2.0 × 10^5^ cells/well in a 24-well dish 24 h before transfection or transduction. XtremeGene9 (Roche) was used at 2 μl/μg DNA to transfect cells with 0.5 μg of both nuclease and green fluorescent protein (GFP) donor constructs. Transductions were performed at an MOI of 5–10 using polybrene. Cells were harvested 72 h after transfection/transduction and read on the flow cytometer. Data were obtained from BFP+ (TLR 2.1) or iRFP+/BFP+ populations (epigenetic TLR).

### Western blot analysis

Reporter 293T cells were collected 72 h after treatment and lysed using radioimmunoprecipitation assay (RIPA) lysis buffer with protease inhibitors, and then lysates were run on a QiaShredder column (Qiagen). Protein concentrations were determined using the BioRad protein assay reagent and 5–10 μg of protein was run on a 10% acrylamide gel alongside Kaleidoscope protein ladder (BioRad) then transferred onto an Immobilo-FL membrane (Millipore) using semidry transfer. The membrane was blocked using Licor blocking buffer before staining with primary rabbit anti-HA (Cell Signaling Technology) and mouse anti-β-actin monoclonal antibodies and secondary PE-conjugated anti-mouse (Medial & Biological Laboratories) and GFP-conjugated anti-rabbit (Invitrogen) monoclonal antibodies. Membrane fluorescence was then detected using the Licor Odyssey.

### Polymerase chain reaction analysis of TAL effector size

Reporter 293T cells were collected 72 h after lentiviral transduction, and genomic DNA was extracted from these cells using the Qiagen Blood and Tissue Kit. Nested polymerase chain reaction (PCR) was performed using two sets of primers to amplify the TAL effector repeats from the integrated megaTAL ORFs as well as the original megaTAL constructs for comparison. The PCR products from the LV-treated samples were cloned into the pJET vector (Clonejet) and transformed into DH5α cells. For each sample, 47 colonies were picked and subjected to colony PCR to amplify the pJET insert. PCR products were run on a 1.2% agarose gel for size determination.

### *In vitro* production of synthetic mRNA

We used the T7-Scribe kit (CellScript) for production of synthetic mRNA. Two different templates were utilized for *in vitro* transcription: a linearized plasmid (megaTAL and HE) or a PCR product containing a T7 promoter (Trex2), generated with the following primers: Trex2 Forward, GGATCCTAATACGACTCACTATAGGGGCCGCCACCATGTCTGAGCCACCTCGGGCTGAG; Trex2 reverse, TTTTTTTTTTTTTTTTTTTTTTTTTTTTTTTTTTTTTTTTTTAGGCTTCGAGGCTTGGAC, with T7 promoter underlined. The resultant mRNA was purified with the RNeasy miniprep columns (Qiagen) and a 5′ cap was enzymatically added using the vaccinia capping system combined with a Cap 2′-O-Methyltransferase enzyme (both from NEB). A poly(A) tailing kit (Life Technologies) was used to add a polyadenylation tail to 5′-capped mRNA and mRNA was purified using the RNeasy kit. The capped/polyadenylated mRNA was eluted in sterile water and stored at −20°.

### Human T-cell culture and transfection

We used the EasySEP Human T-cell enrichment kit (Stemcell technologies) to purify T-cells from human peripheral blood mononuclear cells obtained from anonymous donors at the Puget Sound Blood Center. Isolated T-cells were stimulated with CD3/CD28 activation beads (Life Technologies) based on manufacturer’s instructions. Activated T-cells were cultured in RPMI media supplemented with 10% fetal bovine serum, 1× GlutaMAX, 55µM β−mercaptoethanol, 100 U/ml penicillin, 100 µg/ml streptomycin, 10 mM HEPES and 10 ng/ml IL-2 (Biolegend). The beads were magnetically removed after 36 h of incubation and the T-cell resuspended in fresh culture media for 6–12 h before electroporation (48–56 h total culture time before electroporation). The Neon transfection system (Life Technologies) was used for electroporation, with the 10 µl electroporation kit used for all transfections. T-cells were washed with PBS and resuspended in Buffer T at 2 × 10^7^ cells/ml, with 2 × 10^5^ cells used per transfection. Approximately 1–1.5 µg of each individual mRNA was added to each sample before electroporation. A maximum of 2.5 µg of total mRNA was used for samples that had more than mRNA species (i.e., megaTAL with Trex2). Cells were electroporated with the following conditions: three pulses, 1400 V/pulse, with 10 ms pulse width. After electroporation, cells were immediately dispersed into prewarmed, antibotic-free T-cell media with IL2 and cultured for 4–6 days, with regular media changes, before analysis. Analysis of the T-cell receptor expression was performed using the αCD3-AlexaFluor 488 antibody (Biolegend, clone SK7).

### High-throughput sequencing and analysis

High-throughput sequencing was performed on cells sorted for either BFP expression (293T cells) or which were CD3- (T-cells, except control whole population). PCR primers were designed to target ∼150–200 bp on each side of the putative megaTAL cut site and append Illumina sequencing primer adaptors. Amplicons for all samples including negative controls were then subjected to an additional round of amplification to append library barcodes as well as outermost flowcell sequences. This setup allowed for library indexing at both the barcode and amplicon level. Final libraries were sequenced on 1.25 runs of a MiSeq (version 2) using paired end 250 bp read chemistry with a 9-bp index read. Reads for each de-multiplexed library were aligned to their respective amplicon references using the Phaster read aligner (Phil Green, personal communication) owing to its superior ability to accurately align reads with large indel events. Each aligned read pair was then individually genotyped for the presence of indels. Statistical analysis for indel frequency and skewing (bias toward mn or TALE region) were performed using a permutation analysis (100 000 iterations) and testing against the null hypothesis (negative controls, or lack of skew).

### Statistical analysis

Error bars on graphs represent s.e.m. *P*-values were calculated using Student’s one-tailed unpaired t-test to compare activity of megaTALs with their specific mn counterpart (*P < 0.05 shown as, **P < 0.005, ***P < 0.0005).

## RESULTS

### Designing a TAL effector—mn fusion protein architecture: megaTAL

**Figure 1.**
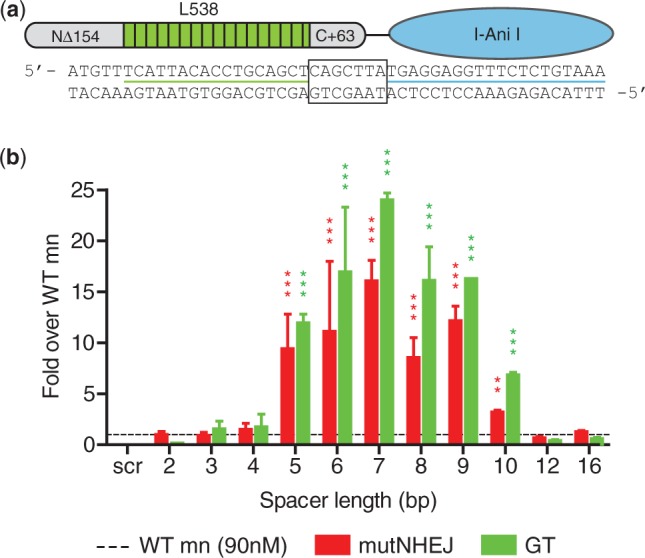
DNA spacer requirement for megaTAL fusions. (**a**) Schematic representation of a megaTAL. The megaTAL architecture involves fusion of a TAL effector with truncated N- and C-terminal domains through a short linker to the N-terminus of a mn (I-AniI, which displays a KD of approximately 90 nM for its cognate target site). Aligned below the megaTAL schematic is the DNA sequence for the L538-I-AniI megaTAL target with a spacer length of 7 bp, the L538 DNA target underlined in green, the I-AniI DNA target underlined in blue and the DNA spacer separating the two outlined with a black box. (**b**) Increase in levels of mutagenic NHEJ (mutNHEJ) and gene targeting (GT) achieved by I-AniI megaTAL fusion. Results shown are derived from assays of cleavage activity using TLR reporter 293T cells treated 72 h with the L538-Zn4-WT megaTAL (mT) or WT I-AniI mn across targets with different spacer lengths or a ‘scrambled’ (scr) I-AniI target. Cleavage activity for L538-WT megaTAL for a given spacer length is normalized to the activity of the WT mn level (represented by a dashed line) across all cell lines. For all graphs, error bars indicate s.e.m and *P*-value comparing megaTALs with their appropriate mn counterpart is indicated by asterisk (*P < 0.05, **P < 0.005, ***P < 0.0005).

Based on the recently proposed DNA binding mechanism of TAL effectors, in which binding is initiated by a series of degenerate TALE repeats that lack sequence specificity, followed by annealing of the sequence-specific RVD array (34–36), we chose to evaluate a TALE-mn fusion architecture (schematized in Figure 1a), hereafter designated as the ‘megaTAL’ architecture. We hypothesized that this architecture would ‘address’ the mn, which has intrinsic specificity for a small set of target sites, to a single desired target, and thus substantially enhance cleavage at that site, without nonspecifically enhancing the activity at related ‘nonaddressed’ targets.

To evaluate the feasibility of this approach, we generated an initial set of megaTALs consisting of a synthetic TAL effector [L538, (37)] fused through a short peptide linker to the mn I-AniI [hereafter WT, (38)], as well as a catalytically inactive I-AniI mutant [hereafter E148D, (20)]. The nuclease activity of each megaTAL was tested in parallel with the corresponding stand-alone I-AniI mn using cell lines containing TLRs with a series of target sites possessing varying spacers between the TAL effector and mn target sites (Supplementary Figure S1a). In these assays, the WT I-AniI megaTAL exhibited up to 18- and 24-fold increases in levels of mutagenic NHEJ (mutNHEJ) and gene targeting, respectively, relative to the stand-alone WT I-AniI (Figure 1b). Importantly, megaTAL activity was found to be spacer dependent, with the maximum increase in activity achieved against a target containing a 7-bp spacer (assuming a 20-bp mn target). No discernible differences in activity were observed between megaTALs constructed with four different protein linkers (Supplementary Figure S1b).

### Effect of TALE array repeat number on megaTAL activity

Because the FokI catalytic domain (used in traditional TALENs) has low intrinsic affinity for DNA and lacks sequence specificity for DNA cleavage (2,5), both the affinity and specificity of a TALEN for an intended target site are entirely dependent on the DNA binding specificity of the TALE RVD arrays, necessitating RVD array lengths of ≥13 TALE repeats to avoid toxicity related to nonspecific binding (19). In contrast, because meganucleases have a high degree of both intrinsic affinity and specificity for a small group of target sites (6,7), we hypothesized that megaTALs could be constructed with much shorter RVD array lengths.

**Figure 2.**
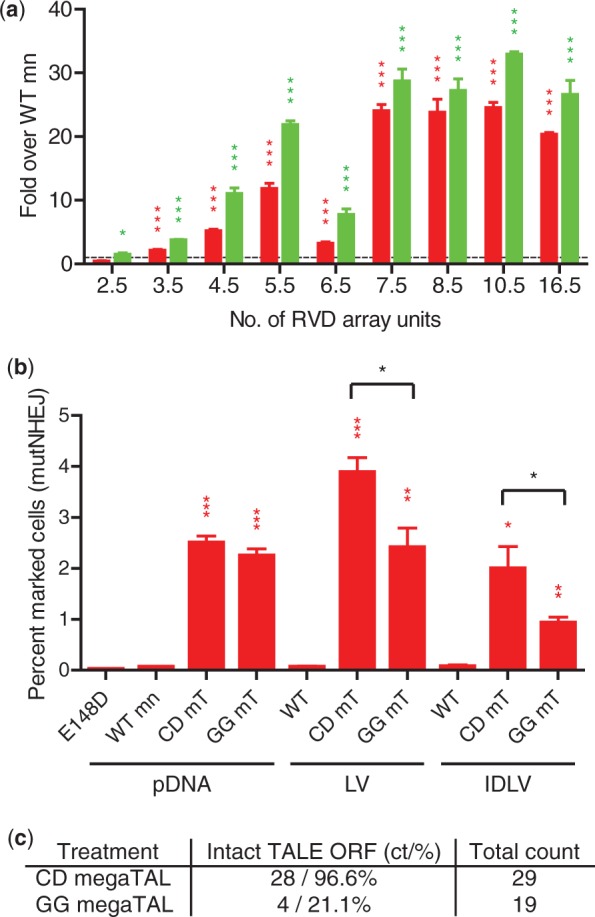
RVD array requirement for megaTAL fusions. (**a**) Level of cleavage activity (assayed by mutNHEJ, shown in red, and gene targeting, shown in green) of megaTALs built with TAL effectors with different number of repeat units. Cleavage activity of TALE-Zn4-WT megaTALs built with a varying number of RVD array units was normalized to the activity of the WT I-AniI mn (represented by a dashed line) across all cell lines tested. (**b**) Level of cleavage activity (assayed by mutNHEJ) of megaTALs built with either a codon diverged or nondiverged 6.5 RVD TAL effector array delivered to reporter cells by DNA transfection or lentiviral delivery. Both the codon diverged (CD) and nondiverged (GG) megaTALs were able to rescue the activity of the WT mn using plasmid DNA (pDNA) transfection, lentiviral (LV) or integration-deficient lentiviral (IDLV) transduction. However, delivery using lentiviral vectors resulted in significantly lower activity in cells treated with the nondiverged version of the megaTAL. (**c**) Proportion of intact (full length) and recombined (short) megaTALs integrated into the genome of 293T cells. Either the codon diverged (CD) or nondiverged (GG) 6.5 RVD WT megaTAL were delivered to cells by lentiviral transduction and the viral integrants were assessed using clonal PCR amplification.

To evaluate the relationship between TALE RVD array length and megaTAL activity, we generated I-AniI megaTALs with truncated versions of the L538 TAL effector ranging from 2.5 to 16.5 repeats long. These megaTALs were tested against their appropriate target, each with a 7-bp spacer (Supplementary Figure S2a), alongside the stand-alone WT I-AniI mn using the TLR reporter lines generated above (Figure 2a, Supplementary Figure S2b). All megaTALs were functional and none exhibited any detectable toxicity, and a megaTAL made with only 3.5 repeats showed a significant increase in levels of both gene targeting and mutNHEJ. Cleavage activity rose in close correlation with the number of TAL effector repeats, with megaTALs made by fusion of a 7.5 RVD TALE with the low affinity WT mn reaching near maximal activity. We observed an idiosyncratic loss of efficacy with the 6.5 RVD TALE, which subsequent analyses demonstrated could be attributed to the interaction between the final RVD and its target base (Supplementary Figure S2c).

By reducing the number of TAL effector repeats present in a megaTAL, we hypothesized that the DNA sequence encoding the TALE domain could be sufficiently diverged so as to reduce its repetitiveness and thus allow lentiviral packaging and delivery of the nuclease transgene without recombination of its open reading frame (ORF). We tested this by constructing a codon diverged megaTAL with a 6.5 RVD array TAL effector (designated CD for ‘codon diverged’, sequence in Supplementary Figure S2d) and comparing its cleavage activity to a nondiverged (designated GG for ‘golden gate’) version using both lentiviral delivery and DNA transfection. A 1.6- and 2.1-fold difference in mutNHEJ levels was observed in reporter cells treated with an lentiviral or integration-deficient lentiviral vector carrying the CD or GG megaTAL version, suggesting a difference in either delivery or expression efficiencies (Figure 2b). Western blot analysis of the megaTAL and mn treated cells revealed expression of the full-length product in diverged megaTAL treated cells, whereas those that received the nondiverged version expressed protein of different sizes (Supplementary Figure 2e). This observation was confirmed using clonal PCR analysis of the TAL effector RVD array from CD and GG megaTAL treated samples, which indicated that 97 and 20% of the integrated ORFs from the respective cell populations encoded megaTAL proteins with intact TALE arrays (Figure 2c).

### Effect of megaTALs on mn target affinity and cleavage activity

The most straightforward explanation for the increased activity observed with the I-AniI megaTAL compared with the stand-alone mn is that the TALE array effectively increases the local concentration of I-AniI by its adjacent cleavage site. We hypothesized that fusing TAL effector DNA binding domains to mns would thus generally act to potentiate the activity of mns toward targets for which they had low affinity, but not toward those for which they displayed slow cleavage kinetics. To test this hypothesis, we further evaluated the behaviors of (i) a series of megaTALs generated from mns with varying affinities for the I-AniI native target, and (ii) a single megaTAL at target sites for which the mn exhibits differing cleavage kinetics.

**Figure 3.**
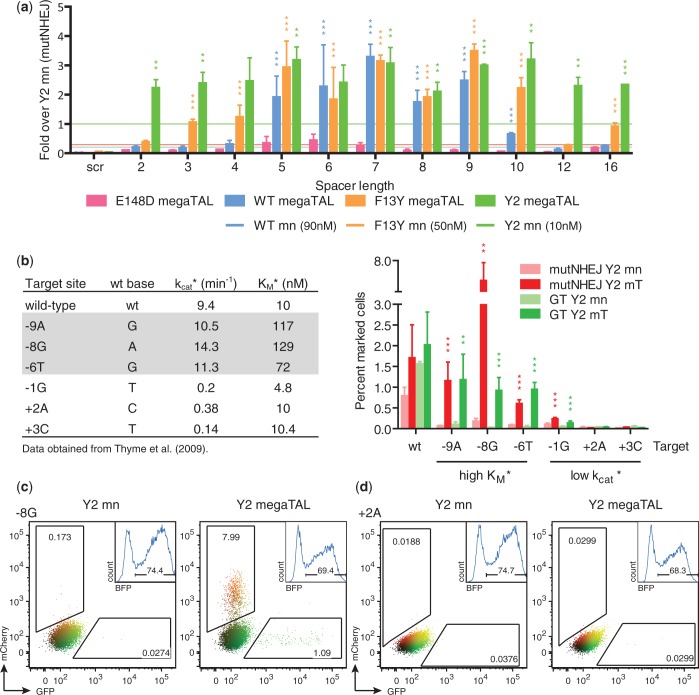
megaTAL addressing acts by increasing the effective mn concentration at the addressed target site. (**a**) Level of cleavage activity (assayed by mutNHEJ) of megaTALs built with mns with varying affinities in TLR cell lines containing targets with different DNA spacer lengths. The level of mutNHEJ for each megaTAL tested was normalized to the activity of Y2 (represented by green line) in the same TLR cell line. The average activity of the WT and F13Y mns across each cell line are represented by blue and orange lines, respectively. (**b**) Effect of megaTAL addressing on activity of Y2 I-AniI toward DNA targets for which it exhibits different biochemical properties. Left panel: Wild-type and singly substituted I-AniI targets and previously determined k_cat_* and K_M_* values. Right panel: Mutagenic NHEJ and gene targeting of Y2 and Y2 megaTAL at each target assayed by TLR. Representative flow plots for data from (b) are shown for the Y2 mn (left column) and megaTAL (right column) against high K_M_* (**c**) and low k_cat_* (**d**) targets. Numbers inside the gates give the percent of mCherry and GFP positive cells and the inset graphs show BFP positive cells on which the experiment was gated.

We generated megaTALs using two additional I-AniI variants (hereafter F13Y and Y2) with slightly increased affinities for the native I-AniI target site (K_D_ = 90 nM, for I-AniI WT; K_D_ = 30 nM, for I-AniI F13Y; K_D_ = 8 nM, for I-AniI Y2) (38). MegaTALs made with the moderate (F13Y) and high (Y2) affinity mn variants also showed significant increases in activity (Figure 3a, Supplementary Figure S1b and c), albeit to a lesser degree (≤13-fold increase for F13Y, ≤4-fold for Y2) than observed with the original I-AniI megaTAL. Thus, the effect of TALE array addressing correlates with the intrinsic affinity of the mn variant: low affinity variants are boosted to a much greater extent than those with intrinsically higher affinity, consistent with the idea that the TAL effector domain influences nuclease activity by increasing the local concentration of the mn cleavage domain at its addressed target site. Additionally, the moderate affinity mn F13Y reached a plateau of activity with a shorter (4.5 RVD) TALE array relative to the TALE array length needed (5.5–7.5 RVD) to maximize the activity of the lower affinity WT I-AniI mn (Supplementary Figure S2b).

We then further defined the TALE ‘addressing’ effect by testing the activity of the high affinity Y2 mn and L538-Y2 megaTAL against a series of DNA target variants that harbor single base-pair substitutions (relative to the native I-AniI target), for which the k_cat_* and K_M_* of the Y2 variant have previously been determined (Figure 3b) (39). Nonnative targets were chosen for which Y2 displays either ‘high K_M_*’ (Y2 exhibits a similar k_cat_* but reduced affinity relative to the original target site) or ‘low k_cat_*’ (Y2 exhibits a reduced k_cat_* but comparable binding affinity compared with the original target site). Cleavage activity was measured using TLR reporter cells with targets composed of the L538 binding site and the individual high K_M_*, low k_cat_* or wt I-AniI targets, separated by an 8-bp spacer. As expected based on its known biochemical properties, the stand-alone Y2 mn showed low or background levels of activity against all six nonnative targets. In contrast, cleavage activity was considerably increased (12–38-fold for HR, 8–27-fold for mutNHEJ) in all high K_M_* targets treated with their corresponding ‘addressed’ megaTAL, while little or no effect was observed for the low k_cat_* targets (0–3-fold for GT and mutNHEJ) (Figure 3b and d). However, megaTAL activity against the low k_cat_* target -1G was found to be significantly increased compared with mn treatment, suggesting that in some cases ‘addressing’ may partially compensate for reduced catalytic activity.

### Increasing on-target cleavage with megaTALs

**Figure 4.**
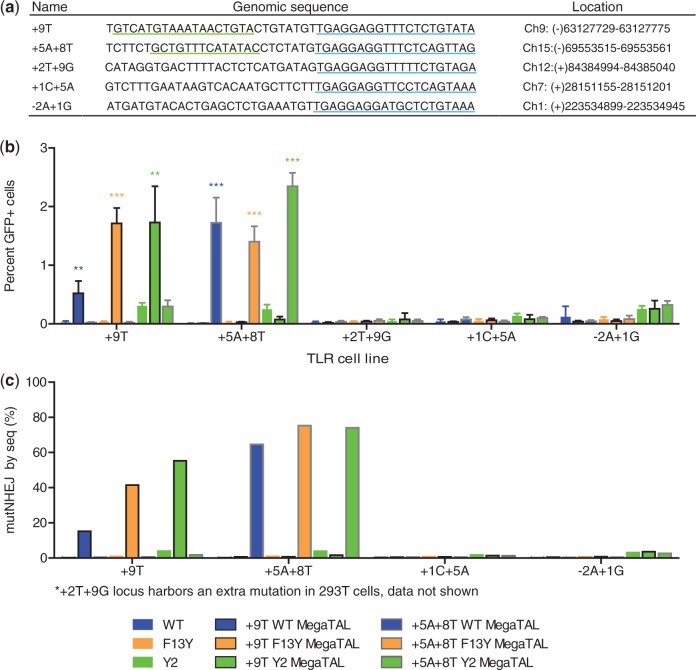
megaTAL addressing selectively increases on-target cleavage. (**a**) I-AniI human genomic near-native sequences and their location in the genome. Synthetic TAL effectors were built against the +9T and +5A+8T sequences underlined in green and the I-AniI near-natives sequences are underlined in blue. (**b**) Level of gene targeting in TLR cells with endogenous human I-AniI near-native targets using ‘addressed’ +9T and +5A+8T megaTALs and ‘unaddressed’ I-AniI mns. mutNHEJ was not measured via TLR due to the presence of a stop codon in some target sites that prevented flow cytometry readout of these events. (**c**) Mutation rates at endogenous targets using ‘addressed’ I-AniI megaTALs and ‘unadressed’ mns. The level of mutNHEJ at the endogenous I-AniI near-native targets in 293T cells after 72 h of treatment with mn and megaTAL variants was determined based on MiSeq sequencing results.

One of the major shortcomings of current nuclease platforms is their potential for off-target cleavage and the negative effects that this may have on the efficacy or safety profile of a putative nuclease-based therapeutic application. To evaluate the specificity properties of the megaTAL architecture, we tested whether a megaTAL could consistently distinguish between ‘addressed’ and ‘nonaddressed’ genomic target sites, all cleaved by a common mn cleavage domain. Near-native sites of the I-AniI target (DNA sequences containing three or fewer base-pair differences from the native I-AniI recognition sequence) were previously identified in the human genome using the UCSC BLAT tool (40). The cleavage activity of the Y2 I-AniI variant mn was assessed at these near-natives targets using both episomal and endogenous targets (data not shown) and the five sites that displayed the highest levels of cleavage by Y2 in those assays were chosen for further examination (Figure 4a). Synthetic TAL effectors specific to regions upstream of the +9T and +5A+8T near-native targets were built and fused to the WT, F13Y and Y2 I-AniI mns. These megaTALs were tested alongside the stand-alone mn variants in TLR reporter cell lines for each of the five targets. The +9T and +5A+8T megaTALs showed increased levels of gene targeting at their addressed targets only, while having no observed effect on the activity at the remaining sites (Figure 4b). Notably, while the addition of a sequence-specific TAL effector domain to the high-affinity Y2 mn did not reduce its cleavage at nonaddressed targets (the Y2 mn and megaTALs show similar levels of GT at off-target sites), treatment with megaTALs made with the lower affinity WT and F13Y mns resulted in levels of gene targeting at addressed targets equivalent to that achieved with the Y2 megaTALs, but without detectable off-target activity.

To verify that a similar effect would be observed at the endogenous near-native loci, 293T cells were treated with +9T megaTALs, +5A+8T megaTALs and mn variants, and the level of mutNHEJ was determined at each near-native site by high-throughput sequencing (Figure 4c, Supplementary Figure S3a and b). These results mirrored those from the reporter assay, with the WT and F13Y megaTALs achieving indel rates up to 75% at their addressed sites, while maintaining near-background activity at nonaddressed sites.

### Highly effective knockout of the T-cell receptor alpha gene in primary T-cells with a megaTAL

To verify that the benefits observed when fusing wild-type mns to a TAL effector would be achievable with an engineered mn and could be captured in a primary cell type in a relevant therapeutic application, we designed a variant of the I-OnuI mn to knockout the T-cell receptor alpha (TCRα) gene. Knockout of the TCRα gene in human T-cells has been proposed as an approach to improve the safety of T-cells endowed with chimeric antigen receptors or heterologous TCRs, by preventing them from generating graft-versus-host disease (41). Thus, knockout of TCRα in human T-cells is among the most translationally relevant applications of rare cleaving nuclease technology.

**Figure 5.**
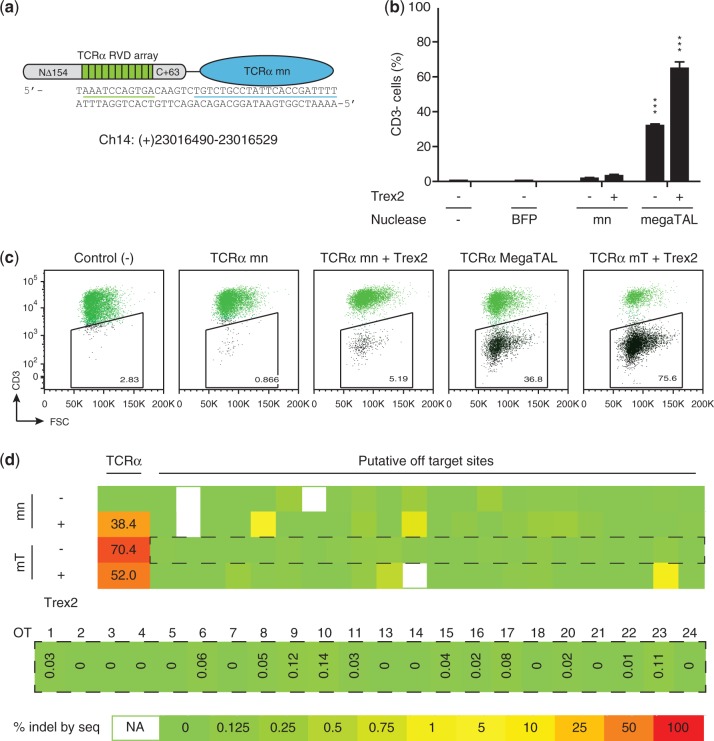
megaTAL targeting TCRα achieves extremely high gene disruption with no detectable off-target cleavage in human primary T-cells. (**a**) Schematic of TCRα megaTAL formed by fusing a synthetic TAL effector built toward the TCRα sequence underlined in green to a designed TCRα mn (target underlined in blue). (**b**) TCR knockout achieved by parental TCRα mn and TCRα megaTAL in human primary T-cells. Human primary T-cells were transfected with 10 micrograms of RNA encoding the indicated constructs, with or without 10 micrograms of mRNA encoding the Trex2 exonuclease. TCRα gene disruption was assessed by measuring the percentage of cells that transition to being CD3- at 5 days following transfection. Mutation rates at the endogenous TCRα gene were also assessed by Miseq sequencing of amplicons around the cleavage site, and showed indel rates >50%, suggesting that there was a preference for cleavage and disruption of the expressed TCRα allele, particularly when the megaTAL is co-expressed with Trex2. (**c**) Representative flow cytometry data from transfection of human primary T-cells with TCRα targeting mn or megaTALs +/− Trex2. (**d**) Heat map showing mutation rates at the endogenous TCRα and putative off-target loci in CD3- primary T-cells treated with the TCRα mn or megaTAL +/− Trex2. Map values were calculated for each locus by subtracting the background rate of indel formation in the control population from that obtained for each nuclease treatment by Miseq sequencing, with the heat map color scale indicated by the key (bottom). Sequencing results across all samples are shown (top), as well as an enlarged view with indel rates at putative off-target (OT) sites in cells treated with the TCRα megaTAL alone (middle). Values calculated as ≤0 are given as ‘0’. Sequencing results were not obtained for putative off-targets OT12 and OT19 and are therefore not shown on the map; other samples for which sequencing results were not obtained are indicated as ‘NA’.

*In vitro* characterization of the TCRα mn showed that its target site affinity and cleavage activity were similar to that of its parent enzyme. Specificity profiling of the TCRα mn and subsequent high-throughput *in vitro* cleavage analysis led to the identification of a number of potential off-target sites, which were cleaved with comparable efficiency to the desired target (Supplementary Figure S4a and e). To determine if generation of a TCRα megaTAL would yield improvements in activity and specificity, the TCRα mn was fused to a 10.5 repeat TALE array designed to bind a DNA sequence upstream of the TCRα mn binding site (Figure 5a). T-cells were then transfected with mRNA encoding either the TCRα megaTAL or the stand-alone mn. In order to increase the efficiency of gene knockout, samples were included wherein either the mn or the megaTAL were co-transfected with mRNA encoding Trex2, a 3′ to 5′ exonuclease that has been shown to significantly increase rates of mutNHEJ at mn cleavage sites (42,43). Disruption of the TCRα gene was measured by staining for CD3, a multi-protein complex that is associated with the α/β T-cell receptor. Strikingly, while disruption of the TCRα gene occurred at minimal rates in cells treated with the stand-alone mn (1.6%), the TCRα megaTAL exhibited a ∼20-fold increase in activity at the addressed target site in the TCRα gene (Figure 5b, representative flow plots are shown in Figure 5c). Co-transfection of each nuclease with Trex2 further increased the rate of disruption, yielding rates of TCRα gene disruption consistently exceeding 70%.

Both on- and off-target cleavage rates were analyzed by sequencing of the TCRα locus and putative off-target sites (identified based on *in vitro* cleavage profiling), in FACS sorted CD3- populations of nuclease/Trex2 treated cells (Figure 5d, Supplementary Figure S5a–c). For each treatment, modification rates at the TCRα locus were expected to be ≥50%, signifying knockout of the gene at the expressing allele in conjunction with some additional rate of modification of the nonexpressing allele. In megaTAL treated cells, we observed 70.4% modification, suggesting a substantial rate (c.a. 1 in 5 cells) of modification of the nonexpressed allele. In contrast, when we expressed the megaTAL with Trex2, we observed that only 52.0% of the alleles carried indels, and even lower rates were obtained from the mn-alone treated cells. We attribute the differences in modification rates observed in the presence and absence of Trex2 to lowered expression of the nuclease in cells co-transfected with the exonuclease. The lower modification rates observed in the low abundance CD3- mn alone treated cells are attributable to contamination of the sorted populations with a larger proportion of background, unstained cells. The sequencing analysis also demonstrated that off-target cleavage in cells treated with the megaTAL alone is extremely low, with only one of 22 potential sites exhibiting a rate of cleavage above background in the presence of Trex2 (OT23). Moreover, the megaTAL produced lower levels of mutNHEJ at several putative off-target loci than the stand-alone mn (i.e. OT18) — this result may reflect statistical variation in the deep sequencing results, or alternatively could be accounted for by reduced expression of the megaTAL nuclease compared with its shorter mn counterpart.

## DISCUSSION

The megaTAL nuclease architecture described here addresses several key limitations of existing nuclease platforms for therapeutic applications, as it enables the generation of compact single chain nucleases that are extremely active and specific.

MegaTALs exhibit dramatic increases in cleavage activity compared with their isolated mn cleavage heads, an effect that appears to be an outcome of the auxiliary TAL effector DNA-binding domain docking (‘addressing’) the mn adjacent to its target and effectively increasing the rate of enzyme–substrate complex formation. Furthermore, the mn-mediated cleavage produces DNA ends with 3′ overhangs that are efficiently processed by the Trex2 exonuclease, thus allowing the co-expression of a megaTAL and Trex2 to achieve unprecedentedly high targeted modification rates in primary cells of >70%.

MegaTALs offer an extreme level of target specificity due to the combined DNA binding and cleavage properties of the protein architecture. The activity of megaTALs is reliant on recognition of the tandem DNA targets of the TALE array and the mn, respectively, effectively extending the length of the target site and reducing the likelihood that more than one target site would be present in the human genome. Similarly, as low affinity mn cleavage heads are not able to efficiently access target sites to which they are not ‘addressed’ by the TALE array, the potential for cleavage at nonaddressed mn recognition sites can be markedly reduced. This is reflected in the megaTAL used for TCRα gene knockout in primary human T-cells, which achieved 70% knockout while above background off-target cleavage was limited to a single site (e.g. Figure 5d, and Supplementary Figure 5). As the single off-target cleavage site identified is in a noncoding region of the genome, this enzyme is of sufficient quality at its present state of development for clinical use. However, a further advantage of the megaTAL architecture is the potential to further ‘tune’ the mn cleavage head to specifically avoid off-target cleavage sites. Thus, we anticipate that iterative refinement of megaTALs cleavage heads will allow the eventual identification of megaTALs that couple high efficiency with perfect on target genomic specificity.

The megaTAL architecture also possesses several properties that facilitate efficient delivery for therapeutic applications. MegaTALs can be expressed with a single ∼2 kb ORF, smaller than two ZFN halves [2.2 kb total (1.1 kb per ZFN half targeting a 9-bp target)] and significantly shorter than a single TALEN half [>5.0 kb total (2.5 kb per TALEN half targeting a 13-bp target)] or Cas9 nuclease (4.1 kb, not including guide RNA). Thus, megaTALs are compatible with efficient vectorization as a DNA, mRNA or with any widely used viral vector system. The capacity to use a TAL effector domain with as few as 5.5 repeats also facilitates codon divergence of the TAL effector repeats to allow packaging into retroviral vectors, where recombination among TAL effector RVDs during reverse transcription can be problematic (25). Finally, the independent function of megaTALs due to their single chain design avoids the possibility of inappropriate heterodimerization that may occur with enzymes based on dimerization-dependent cleavage domains, such as FokI, an important advantage for applications requiring multiplex genetic modifications.

## SUPPLEMENTARY DATA

Supplementary Data are available at NAR Online, including [44].

## FUNDING

National Institutes of Health [RL1CA133832, UL1DE019582, R01-HL075453, PL1-HL092557, RL1-HL092553, RL1-HL92554 and U19-AI96111]; Seattle Children's Center for Immunity and Immunotherapies. Funding for open access charge: NIH [U19-AI96111].

*Conflict of interest statement*. A.M.S. is an employee of Cellectis therapeutics, and receives salary and equity compensation. He is also founder, member of the board of directors and equity holder in Pregenen Inc. J.J. is an employee of Pregenen Inc., and receives salary and equity compensation. A.A. is an employee of Pregenen Inc., and receives salary and equity compensation. P.D. is an employee of Cellectis therapeutics, and receives salary and equity compensation. A.G. is an employee of Cellectis therapeutics, and receives salary and equity compensation.

## Supplementary Material

Supplementary Data
