# Supplementary material for: megaTALs: a rare-cleaving nuclease architecture for therapeutic genome engineering
Source: Nucleic Acids Res. 2013 Nov 26;42(4):2591–601. doi: 10.1093/nar/gkt1224 (PMC3936731; doi:10.1093/nar/gkt1224)
Supplement: Supplementary Data [file supp_42_4_2591__index.html]

megaTALs: a rare-cleaving nuclease architecture for therapeutic genome engineering — megaTALs: a rare-cleaving nuclease architecture for therapeutic genome engineering — Supplementary Data 

# megaTALs: a rare-cleaving nuclease architecture for therapeutic genome engineering

## Supplementary Data

files

**Files in this Data Supplement:**

- Supplementary Data - pdf file
