# Supplementary material for: megaTALs: a rare-cleaving nuclease architecture for therapeutic genome engineering
Source: Nucleic Acids Res. 2013 Nov 26;42(4):2591–601. doi: 10.1093/nar/gkt1224 (PMC3936731; doi:10.1093/nar/gkt1224)
Supplement: Supplementary Data [file supp_gkt1224_nar-02895-f-2013-File007.pdf]

Supplementary Information

megaTALs: a rare-cleaving nuclease architecture for therapeutic genome engineering

Sandrine J. Boissel, Jordan Jarjour, Alexander Astrakhan, Andrew Adey, Jay Shendure, Barry Stoddard, Michael Certo, David Baker, and Andrew M. Scharenberg

CONTENTS

Supplementary Methods

Supplementary Figures

- *Supplementary Figure 1. Comparison of cleavage activity of megaTALs made with different protein linkers and meganuclease variants with varying affinity in 293T TLR cells containing targets with different DNA spacer lengths*
- *Supplementary Figure 2. Comparison of cleavage activity of megaTALs made with different numbers of RVD array units in 293T TLR cells*
- *Supplementary Figure 3. Cleavage activity of "addressed" and "unaddressed" megaTALs using both 293T TLR cells and high-throughput sequencing at endogenous DNA loci in primary human T-cells*
- *Supplementary Figure 4. Characterization of the TCRα meganuclease and in vitro analysis of on-target and putative off-target cleavage*
- *Supplementary Figure 5. High-throughput sequencing results and analysis of on-target and putative off-target cleavage in T-cells*
- *Supplementary Figure 6. Coding sequences of a megaTAL, meganuclease variants and RVD array tested*

SUPPLEMENTARY METHODS

DNA binding and cleavage flow cytometry assays

Measurement of the DNA binding affinities and cleavage activities of meganucleases with various DNA substrates was performed as described (20). Briefly, *Saccharomyces cerevisiae* strain EBY100 was transfected with the pCTCON2 surface display constructs encoding the indicated LHEs as fusions with the Aga2 protein under the control of the inducible $Gal_{1\text{-}10}$ promoter. For DNA binding affinity assays, samples of 10^5 induced yeast were incubated with the indicated concentrations of Alexa-647 labeled double-stranded DNA substrates in binding buffer (150 mM KCl, 10 mM NaCl, 10 mM HEPES, 5 mM K-glutamate, 5 mM $CaCl_2$, 0.05% BSA, pH 8.0) for 2 hours at 4° C and co-stained with FITC-labeled anti-Myc antibody (Immunology Consultants Laboratories) used at 1:200 dilution. Cells were washed 3 times with cold binding buffer and analyzed. Median Alexa-647 values from Myc epitope normalized sub-populations were used to calculate binding dissociation constants. For DNA cleavage assays, samples of 10^5 yeast were stained with biotinylated anti-HA antibody (Covance) used at 1:300 dilution in staining buffer (180 mM KCl, 10 mM NaCl, 10 mM HEPES, 0.1% BSA, pH 7.0) for 30 minutes on ice, washed twice with cold conjugation buffer (600 mM KCl, 10 mM NaCl, 10 mM HEPES, 0.1% BSA, pH 7.0) then counter-stained with pre-conjugates of dual Alexa-647/biotin labeled DNA substrates and streptavidin-PE for 10 minutes on ice. Cells were then washed once with conjugation buffer and once with binding buffer, then split into control and experimental samples and placed in pre-warmed binding buffer containing either 5 mM $CaCl_2$ or 5 mM $MgCl_2$ respectively. The reaction was allowed to proceed for 20 minutes, then quenched with cold staining buffer and kept on ice until analysis by flow cytometry. Approximately 10,000 cells were acquired from each sample in the binding and cleavage assays using an LSRII flow cytometer (Beckton Dickenson).

SUPPLEMENTARY FIGURES

S1a

Spacer (bp)	Target site
2	TACACATGTACACGTTCATTACACCTGCAGCTTATGAGGAGGTTTCTCTGTAAA
3	TACACATGTACACTTCATTACACCTGCAGCTTTATGAGGAGGTTTCTCTGTAAA
4	TACACATGTACATTCATTACACCTGCAGCTGTTATGAGGAGGTTTCTCTGTAAA
5	TACACATGTACTTCATTACACCTGCAGCTCGTTATGAGGAGGTTTCTCTGTAAA
6	TACACATGTATTCATTACACCTGCAGCTACGTTATGAGGAGGTTTCTCTGTAAA
7	TACACATGTTTCATTACACCTGCAGCTCAGCTTATGAGGAGGTTTCTCTGTAAA
8	TACACATGTTCATTACACCTGCAGCTACAGCTTATGAGGAGGTTTCTCTGTAAA
9	TACACATTTCATTACACCTGCAGCTATCAGCTTATGAGGAGGTTTCTCTGTAAA
10	TACACATTCATTACACCTGCAGCTGATCAGCTTATGAGGAGGTTTCTCTGTAAA
12	TACATTCATTACACCTGCAGCTATGATCAGCTTATGAGGAGGTTTCTCTGTAAA
16	TTCATTACACCTGCAGCTACACATGATCAGCTTATGAGGAGGTTTCTCTGTAAA

S1b

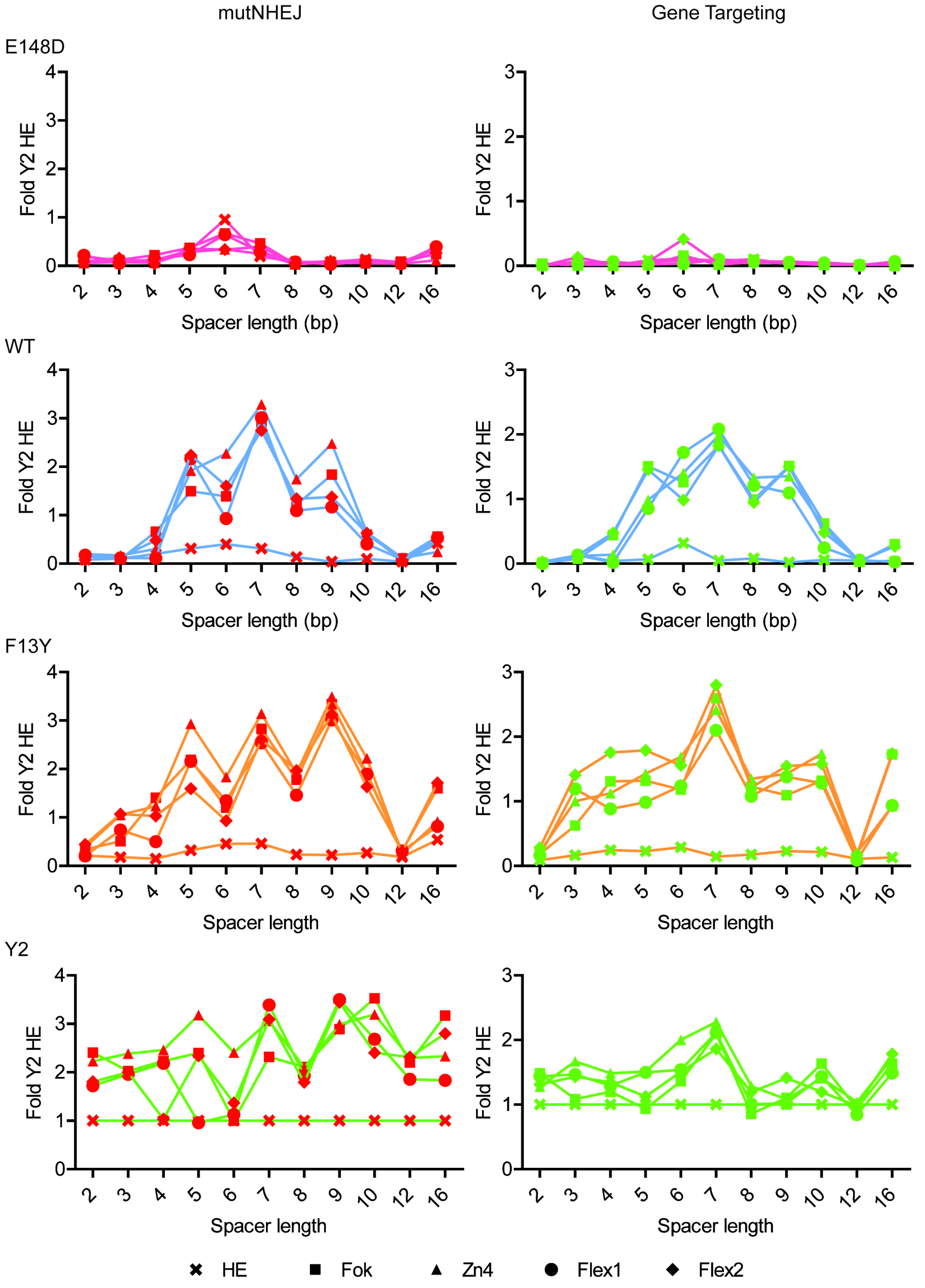
mutNHEJ
Gene Targeting
E148D
WT
F13Y
Y2
Fold Y2 HE
Spacer length (bp)
Spacer length
0
1
2
3
4
2
3
4
5
6
7
8
9
10
12
16
HE
Fok
Zn4
Flex1
Flex2

S1c

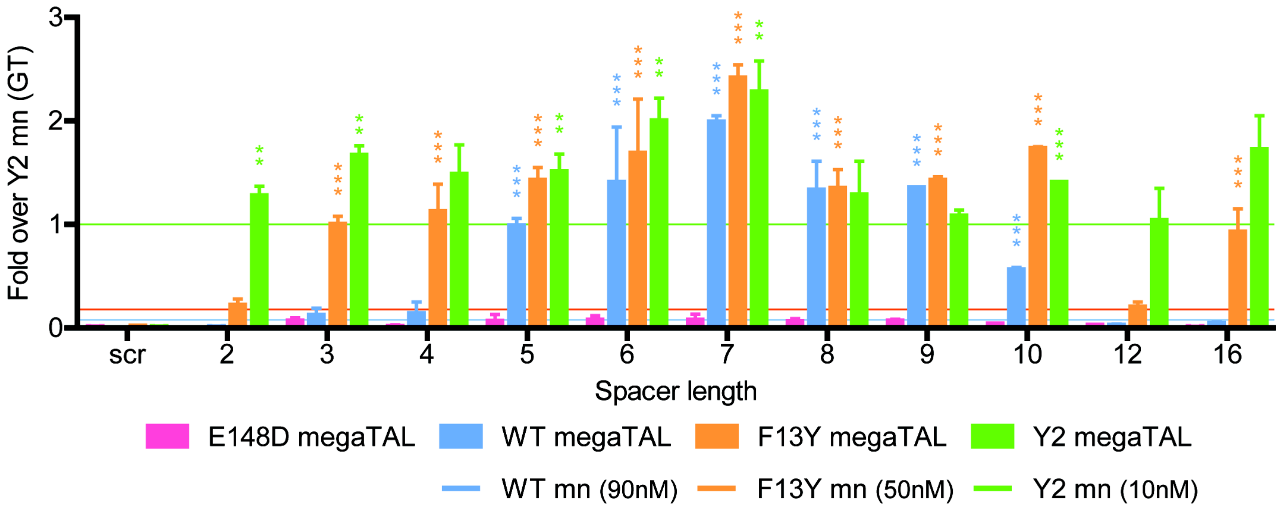

Supplementary Figure 1. Comparison of cleavage activity of megaTALs made with different protein linkers and meganuclease variants with varying affinity in 293T TLR cells containing targets with different DNA spacer lengths

(**a**) Targets with varying DNA spacers (2-16 nucleotides) separating the L538 TAL effector (underlined green) and I-AniI (underlined blue) binding sites tested in the Traffic Light Reporter assay for cleavage activity with megaTALs and their corresponding standalone meganucleases. (**b**) Level of mutNHEJ (red symbols, left column) and gene targeting (green symbols, right column) measured in Traffic Light reorter cell lines with targets from (a). Reporter cells were treated with megaTALs made by fusing the L538 TAL effector to either the E148D (pink lines), WT (blue lines), F13Y (orange lines) or Y2 (green lines) variant of the I-AniI meganuclease by four different protein linkers (Fok, Zn4, Flex1 and Flex2). Activity of the "naked" meganuclease variant was also tested. (**c**) Level of gene targeting for megaTALs built with all four I-AniI variants across TLR targets from (a) in 293T cells.

S2a

No. of RVDs	Target site
2.5	ACATTCATTACACCTGCAGCTGTTATGAGGAGGTTTCTCTGTAAA
3.5	ACTTCATTACACCTGCAGCTCGTTATGAGGAGGTTTCTCTGTAAA
4.5	ATTCATTACACCTGCAGCTACGTTATGAGGAGGTTTCTCTGTAAA
5.5	TTCATTACACCTGCAGCTCAGCTTATGAGGAGGTTTCTCTGTAAA
6.5	TCATTACACCTGCAGCTACAGCTTATGAGGAGGTTTCTCTGTAAA
7.5	CATTACACCTGCAGCTATCAGCTTATGAGGAGGTTTCTCTGTAAA
8.5	ATTACACCTGCAGCTGATCAGCTTATGAGGAGGTTTCTCTGTAAA
10.5	TACACCTGCAGCTATGATCAGCTTATGAGGAGGTTTCTCTGTAAA
16.5	TTCATTACACCTGCAGCTCAGCTTATGAGGAGGTTTCTCTGTAAA

S2b

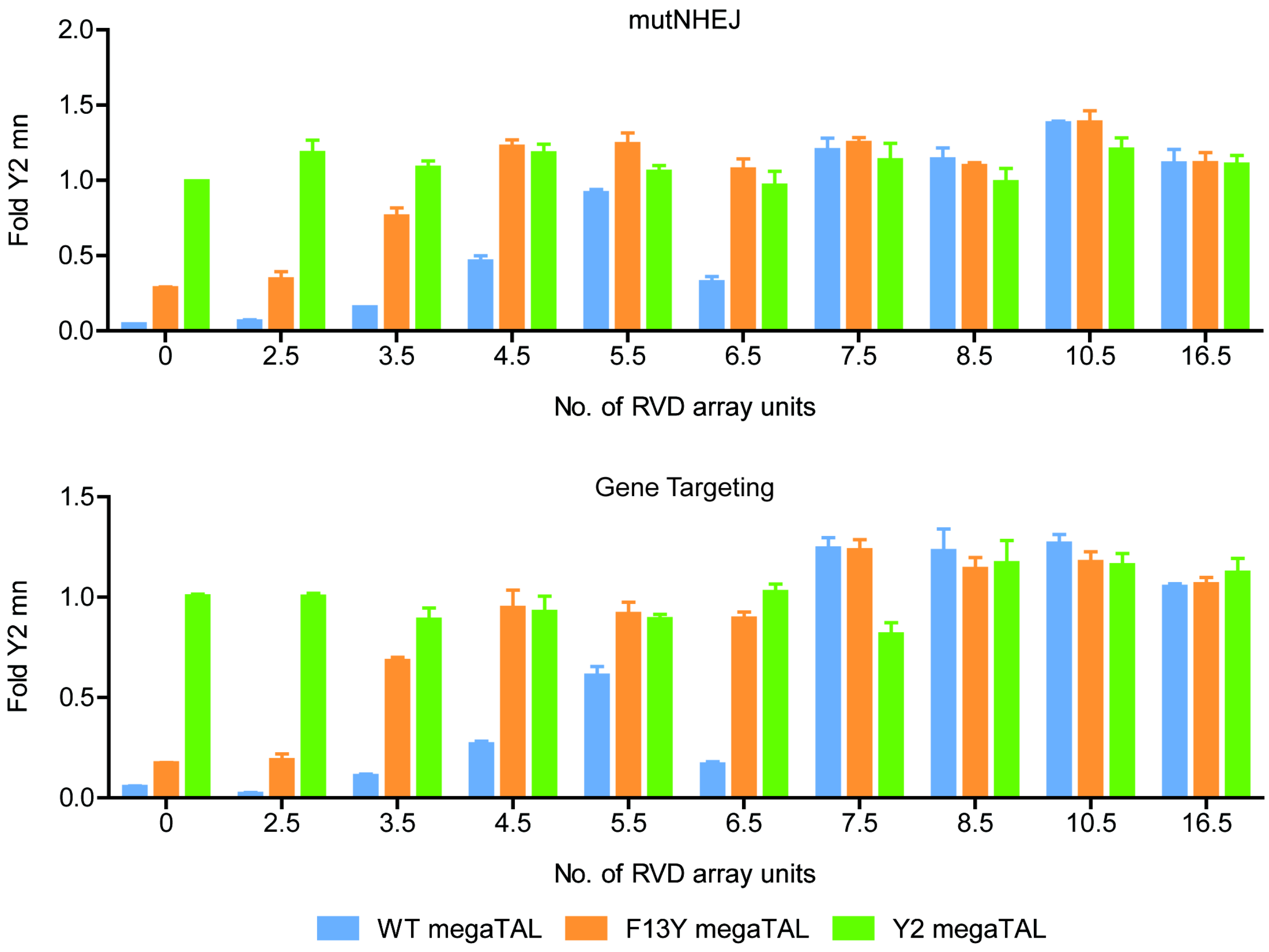

S2c

Name	Last RVD	Target site
A	NI	TCATTACACCTGCAGCTACAGCTTATGAGGAGGTTTCTCTGTAAA
C	HG	TCATTACACCTGCAGCTCCAGCTTATGAGGAGGTTTCTCTGTAAA
G	NN	TCATTACACCTGCAGCTGCAGCTTATGAGGAGGTTTCTCTGTAAA
T	NG	TCATTACACCTGCAGCTTCAGCTTATGAGGAGGTTTCTCTGTAAA
A*	NI	TCATTACACCTGCAGCTAGCGCTTATGAGGAGGTTTCTCTGTAAA

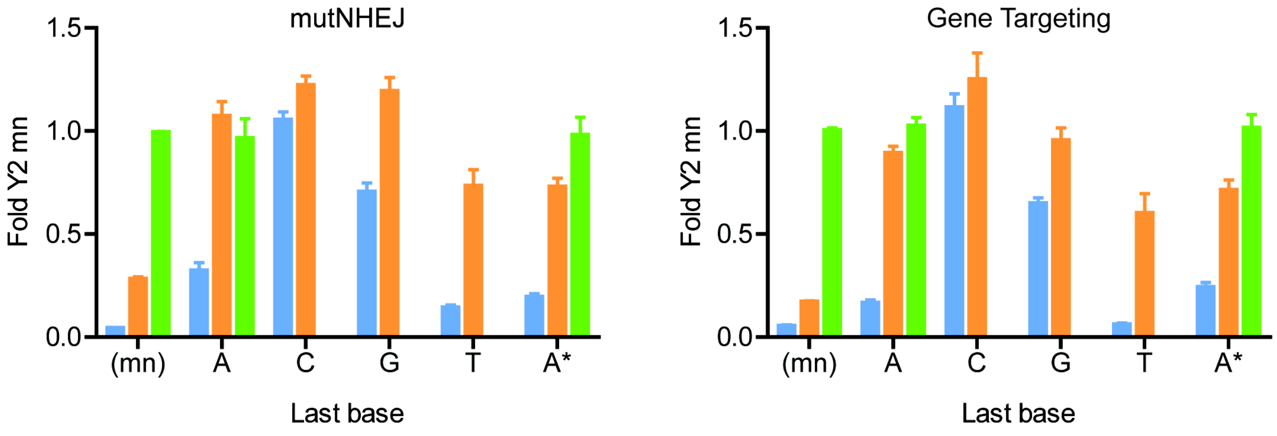

S2d

6.5(C) RVD array CD

```
      L  T  P  D  Q  V  V  A  I  A  S  X  X  G  G  K  Q  A  L  E  T  V  Q  R  L  L  P  V  L  C  Q  D  H  G
NN   CTCACACCTGACCAAGTTGTGGCAATTGCAAGCAATAACGGCGGCAAACAGGCACTCGAAACTGTGCAGCGGCTGCTCCCAGTGCTCTGCCAGGATCACGGG
HD   CTCACTCCCGATCAGGTCGTCGCTATTGCCTCTCATGACGGGGGGAAACAAGCTCTTGAAACAGTCCAGAGGCTTCTCCCTGTTCTGTGCCAAGATCATGGC
NI   TTGACCCCCGACCAGGTTGTTGCCATAGCCTCCAATATCGGAGGAAAACAGGCTCTGGAAACCGTCCAAAGACTCCTTCCCGTCTTGTGCCAGGACCATGGA
NN   CTTACCCCTGATCAAGTGGTGGCCATCGCTAGCAACAATGGGGGCAAGCAGGCCCTGGAGACCGTGCAACGGCTCCTCCCCGTTCTCTGTCAAGACCATGGG
HD   CTGACACCAGACCAAGTCGTTGCTATCGCCAGCCACGATGGAGGTAAGCAAGCACTTGAGACAGTTCAGAGACTGTTGCCTGTGCTGTGTCAGGACCACGGC
NG   CTGACTCCAGACCAGGTGGTCGCAATCGCATCCAATGGCGGAGGGAAGCAGGCTTTGGAAACGGTTCAAAGGCTGCTGCCAGTCCTTTGCCAAGACCACGGA
HD   TTGACTCCTGATCAGGTAGTTGCAATAGCTTCTCACGACGGCGGGAAACAAGCACTCGAG
```

6.5(C) RVD array GG

```
      L  T  P  D  Q  V  V  A  I  A  S  X  X  G  G  K  Q  A  L  E  T  V  Q  R  L  L  P  V  L  C  Q  D  H  G
NN   CTGACCCCGGACCAAGTGGTGGCTATCGCCAGCAACAATGGCGGCAAGCAAGCGCTCGAAACGGTGCAGCGGCTGTTGCCGGTGCTGTGCCAGGACCATGGC
HD   CTGACTCCGGACCAAGTGGTGGCTATCGCCAGCCACGATGGCGGCAAGCAAGCGCTCGAAACGGTGCAGCGGCTGTTGCCGGTGCTGTGCCAGGACCATGGC
NI   CTGACCCCGGACCAAGTGGTGGCTATCGCCAGCAACATTGGCGGCAAGCAAGCGCTCGAAACGGTGCAGCGGCTGTTGCCGGTGCTGTGCCAGGACCATGGC
NN   CTGACCCCGGACCAAGTGGTGGCTATCGCCAGCAACAATGGCGGCAAGCAAGCGCTCGAAACGGTGCAGCGGCTGTTGCCGGTGCTGTGCCAGGACCATGGC
HD   CTGACTCCGGACCAAGTGGTGGCTATCGCCAGCCACGATGGCGGCAAGCAAGCGCTCGAAACGGTGCAGCGGCTGTTGCCGGTGCTGTGCCAGGACCATGGC
NG   CTGACCCCGGACCAAGTGGTGGCTATCGCCAGCAACGGTGGCGGCAAGCAAGCGCTCGAAACGGTGCAGCGGCTGTTGCCGGTGCTGTGCCAGGACCATGGC
HD   CTGACCCCGGACCAAGTGGTGGCTATCGCCAGCCACGATGGCGGCAAGCAAGCGCTCGAG
```

S2e

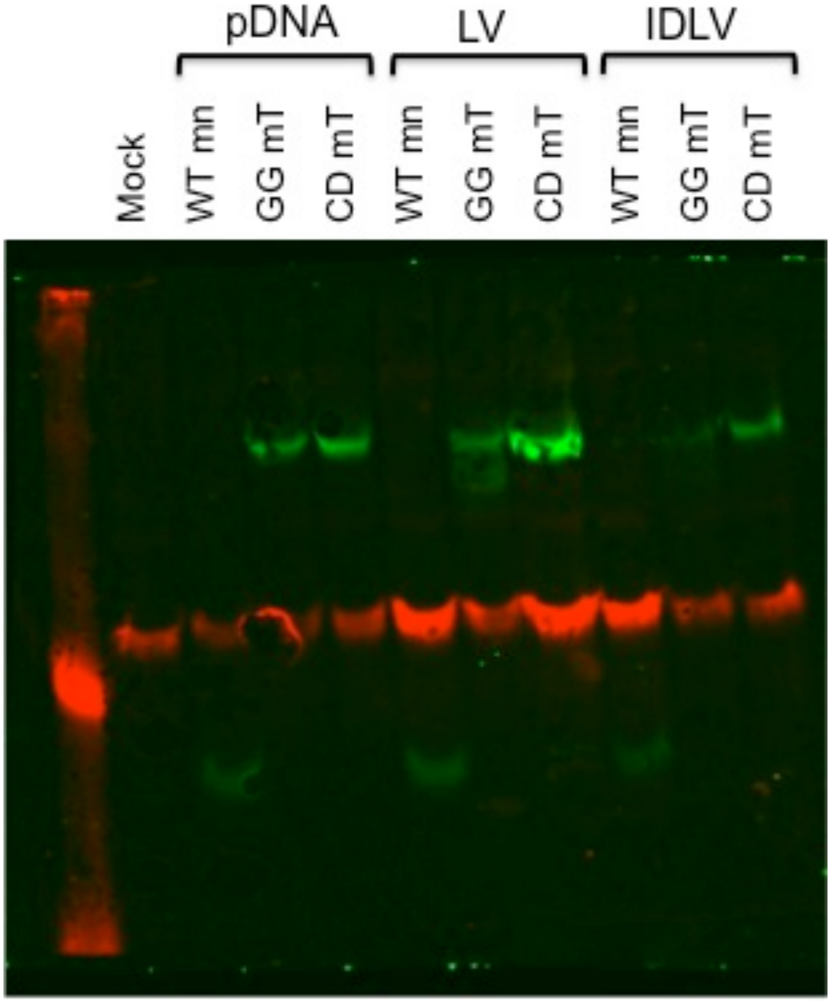

Supplementary Figure 2. Comparison of cleavage activity of megaTALs made with different numbers of RVD array units in 293T TLR cells

(**a**) Traffic Light Reporter targets used to test the effect of number of TAL effector array units on megaTAL activity, with the TAL effector and I-AniI binding sites underlined in green and blue, respectively. (**b**) Level of mutNHEJ (top) and gene targeting (bottom) in Traffic Light reporter cells using WT, F13Y and Y2 I-AniI megaTALs with varying number of TAL effector repeat units (2.5-16.5) or the standalone meganuclease (0). (**c**) Testing of the RVD array requirement of megaTALs (**Figure 2a**) revealed some idiosyncratic variations in activity - the 6.5 repeat I-AniI

megaTAL exhibited a consistent reduction in activity relative to other megaTALs. We experimentally evaluated two potential explanations for thie reduced activity of the 6.5 repeat I-AniI megaTAL – a cryptic binding target for the 6.5 repeat TALE (TACAGCTT) within the TALE-spacer region of the intended target, and the use of two low affinity RVDs (NG for T and NI for A) at the C-terminus of the TAL effector. Results from these analyses indicate that the identity of the last RVD and nucleotide pair can have an important effect on cleavage activity, while the presence of a possible cryptic TALE binding site did not significantly impact activity. Top panel: Traffic light reporter target sites for the 6.5 repeat megaTALs tested against targets in which the last base of the TAL effector target is varied (A, C, G and T) or the second cryptic TALE binding site ablated (A*). The TAL effector and I-AniI binding sites of the target are underlined in green and blue respectively, and the identity of the last RVD residues given. Bottom panel: Level of mutNHEJ (left) and gene targeting (right) in Traffic Light Reporter cells using WT, F13Y and Y2 I-AniI megaTALs with 6.5 repeat TAL effectors varying in their last RVD or the standalone meganuclease(mn).

(**d**) DNA sequence of the codon diverged (CD, top) and non-diverged (GG, bottom) 6.5 RVD TAL effector array used to test lentiviral packaging. Sequences of each repeat are aligned with alternate codons colored red and black and RVD codons shown in bold.

(**e**) Western blot of protein samples obtained from reporter cells treated with the WT meganuclease or 6.5 RVD codon diverged (CD) or non-diverged (GG) WT megaTAL by plasmid DNA (pDNA), lentiviral (LV) or integration-deficient lentiviral (IDLV) delivery. Detection of HA-tagged nucleases and actin protein are pseudo-colored green and red, respectively.

S3a

Total sequencing results for I-AniI near-native genomic loci				
Locus	Nuclease treatment	Total read pairs (ct.)	Reads with indels (ct.)	mutNHEJ (%)
+9T	Control	42580	59	0.139
	WT HE	39566	93	0.235
	F13Y HE	17783	151	0.849
	Y2 HE	19825	783	3.95
	+9T WT MT	23162	3529	15.236
	+9T F13Y MT	19749	8180	41.42
	+9T Y2 MT	49186	27184	55.268
	+5A+8T WT MT	23205	100	0.431
	+5A+8T F13Y MT	21200	139	0.656
	+5A+8T Y2 MT	40611	714	1.758
+5A+8T	Control	24951	54	0.216
	WT HE	21441	43	0.201
	F13Y HE	11783	112	0.951
	Y2 HE	12007	462	3.848
	+9T WT MT	16162	104	0.643
	+9T F13Y MT	17023	120	0.705
	+9T Y2 MT	26320	429	1.63
	+5A+8T WT MT	13674	8838	64.634
	+5A+8T F13Y MT	16656	12542	75.3
	+5A+8T Y2 MT	22969	17013	74.069
+1C+5A	Control	36732	93	0.253
	WT HE	29690	87	0.293
	F13Y HE	21497	162	0.754
	Y2 HE	19125	326	1.705
	+9T WT MT	21961	106	0.483
	+9T F13Y MT	26719	167	0.625
	+9T Y2 MT	39260	546	1.391
	+5A+8T WT MT	22172	129	0.582
	+5A+8T F13Y MT	24336	131	0.538
	+5A+8T Y2 MT	29714	386	1.299

Total sequencing results for I-AniI near-native genomic loci, *cont.*

Locus	Nuclease treatment	Total read pairs (ct.)	Reads with indels (ct.)	mutNHEJ (%)
-2A+1G	Control	44132	70	0.159
	WT HE	24655	29	0.118
	F13Y HE	25907	153	0.591
	Y2 HE	20078	625	3.113
	+9T WT MT	18217	77	0.423
	+9T F13Y MT	23844	197	0.826
	+9T Y2 MT	40806	1470	3.602
	+5A+8T WT MT	22254	89	0.4
	+5A+8T F13Y MT	18187	89	0.489
	+5A+8T Y2 MT	43726	1148	2.625

S3b

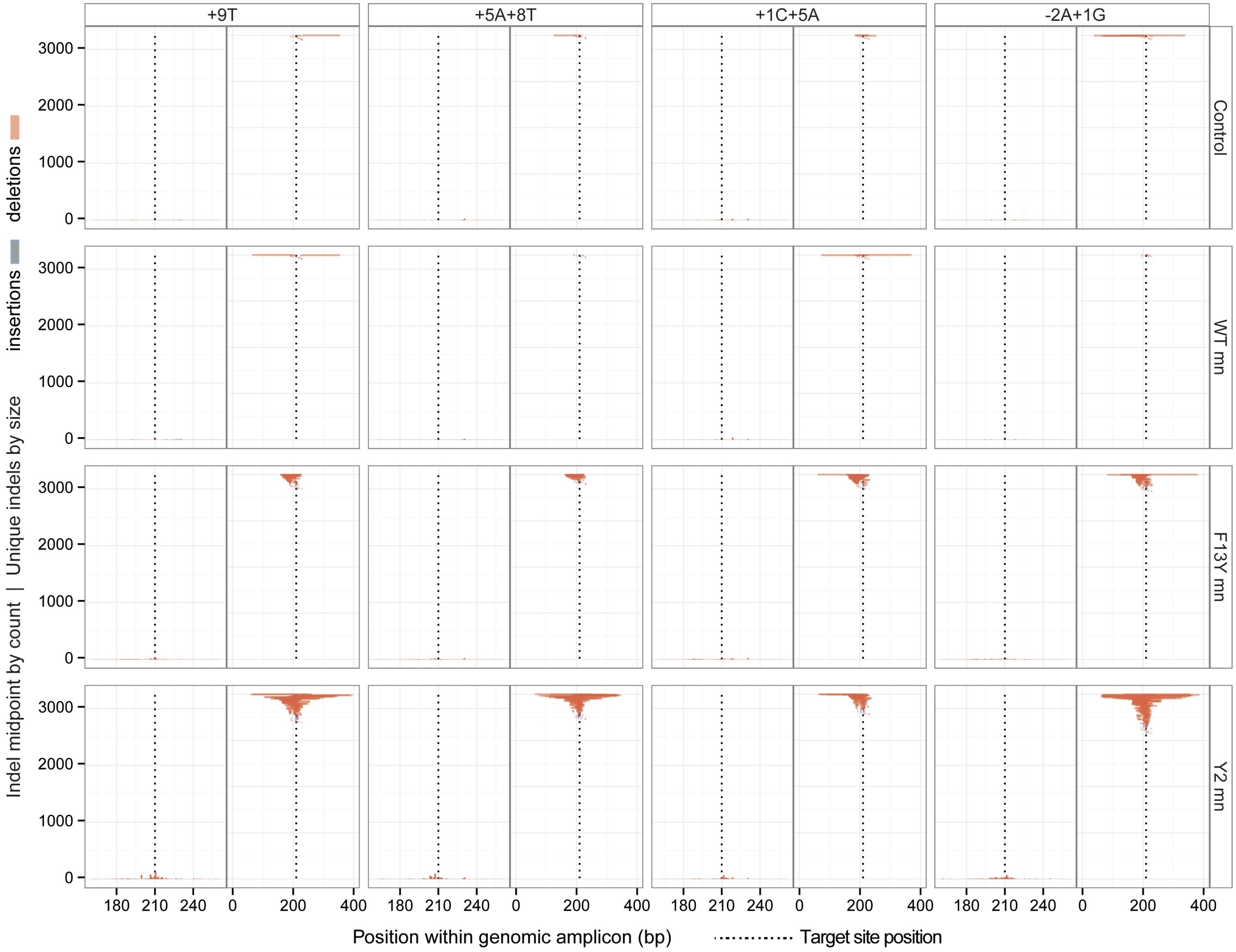

+9T
+5A+8T
+1C+5A
-2A+1G
Control
WT mn
F13Y mn
Y2 mn
deletions
insertions
Indel midpoint by count | Unique indels by size
3000
2000
1000
0
180 210 240
0 200 400
Position within genomic amplicon (bp)
Target site position

S3b, *cont.*

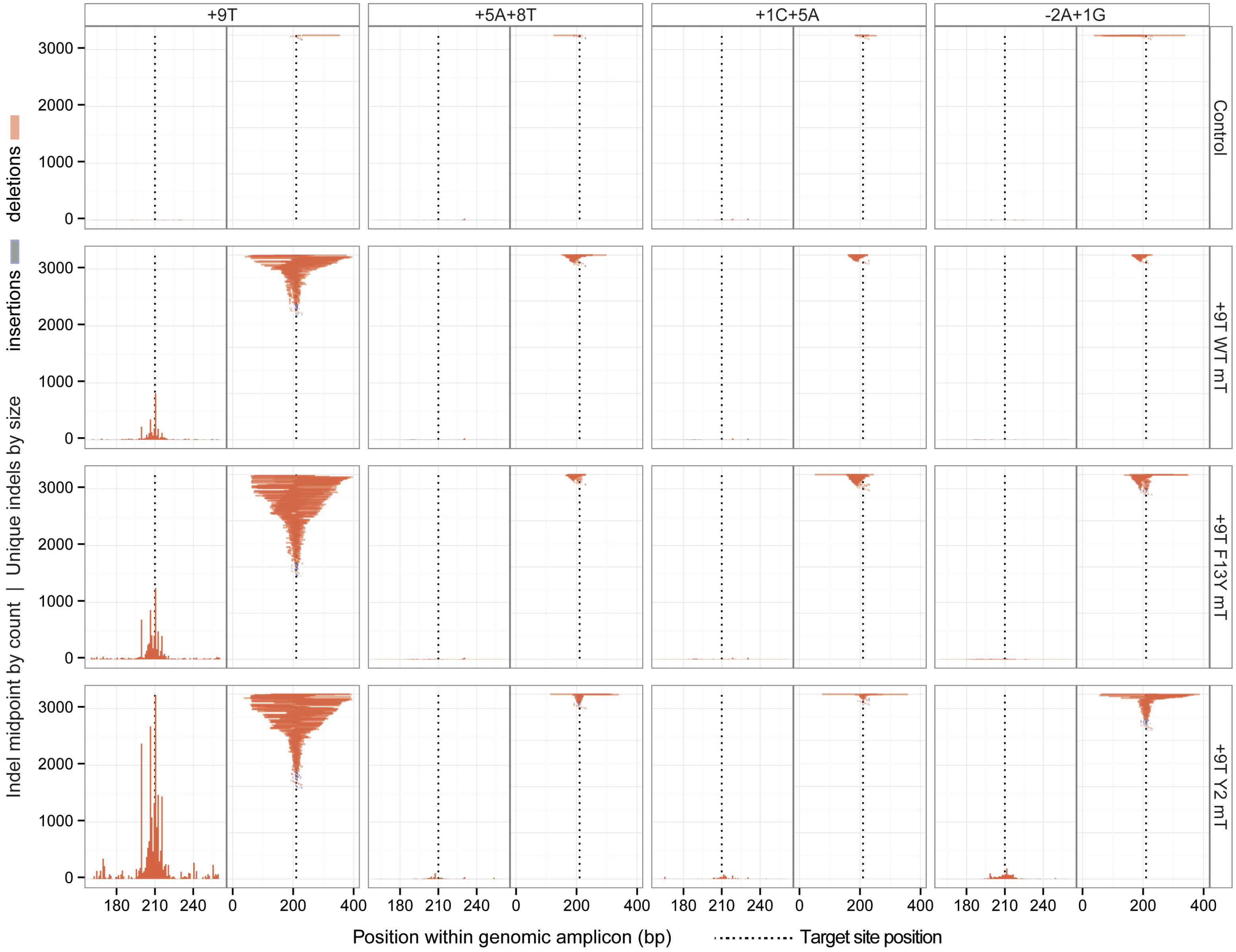

+9T
+5A+8T
+1C+5A
-2A+1G
Control
+9T WT mT
+9T F13Y mT
+9T Y2 mT
deletions
insertions
Indel midpoint by count | Unique indels by size
0
1000
2000
3000
180 210 240
0 200 400
Position within genomic amplicon (bp)
Target site position

S3b, *cont.*

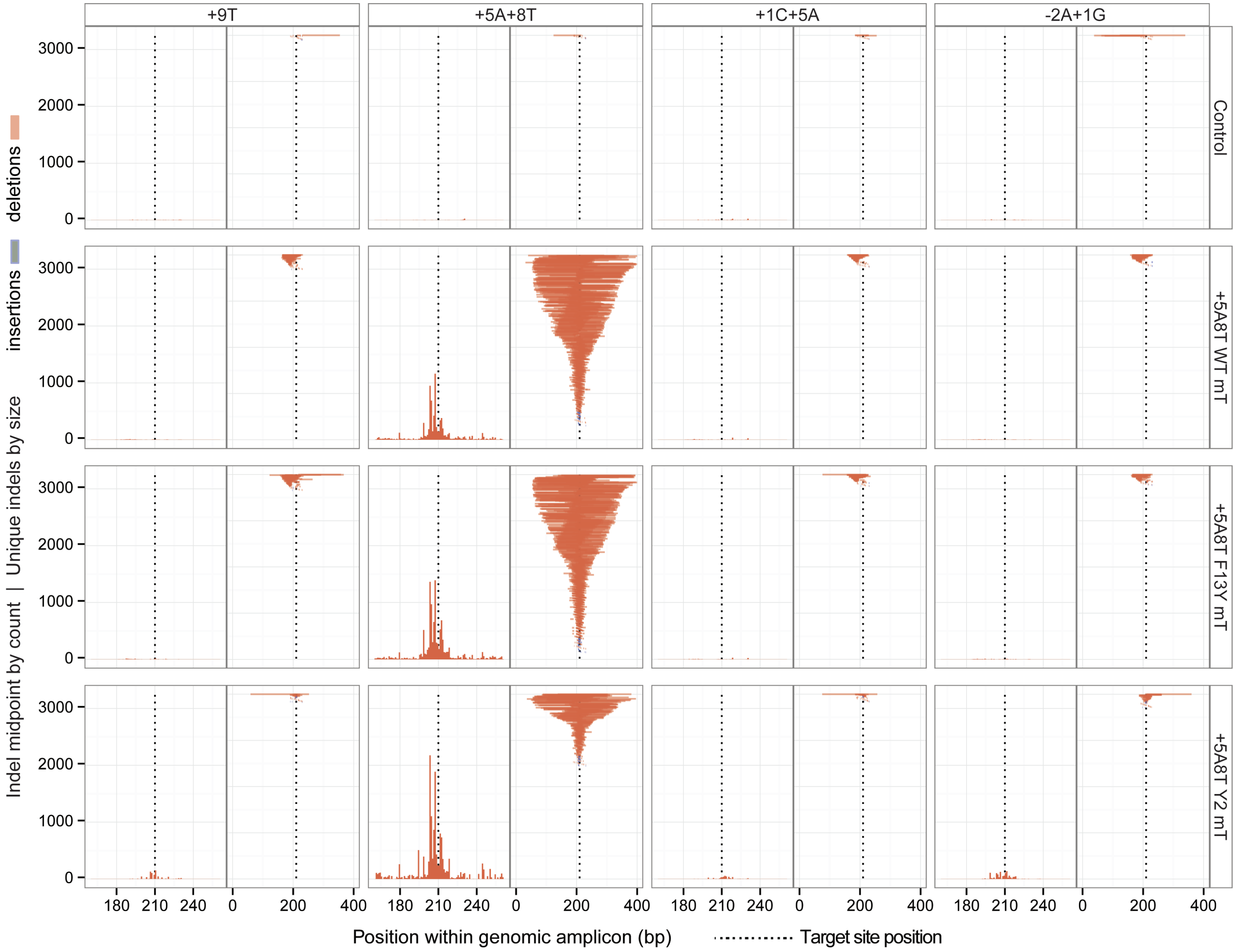

+9T
+5A+8T
+1C+5A
-2A+1G
Control
+5A8T WT mT
+5A8T F13Y mT
+5A8T Y2 mT
deletions
insertions
Indel midpoint by count | Unique indels by size
3000
2000
1000
0
180 210 240
0 200 400
Position within genomic amplicon (bp)
Target site position

Supplementary Figure 3. Cleavage activity of “addressed” and “unaddressed” megaTALs using both 293T TLR cells and high-throughput sequencing at endogenous DNA loci in primary human T-cells

(**a**) High-throughput sequencing results at endogenous human I-AniI near-native targets in 293T cells by nuclease treatment listing full Miseq run statistics. (**b**) Plots showing the position and size of indels found by high-throughput sequencing of megaTAL and meganuclease treated 293T cells at “addressed” and “unaddressed” loci. For each loci, the panel on the left shows a histogram of indels by their midpoint position and the panel on the right shows a tornado plot of all unique indels that were identified, sorted by insertion or deletion size.

S4a

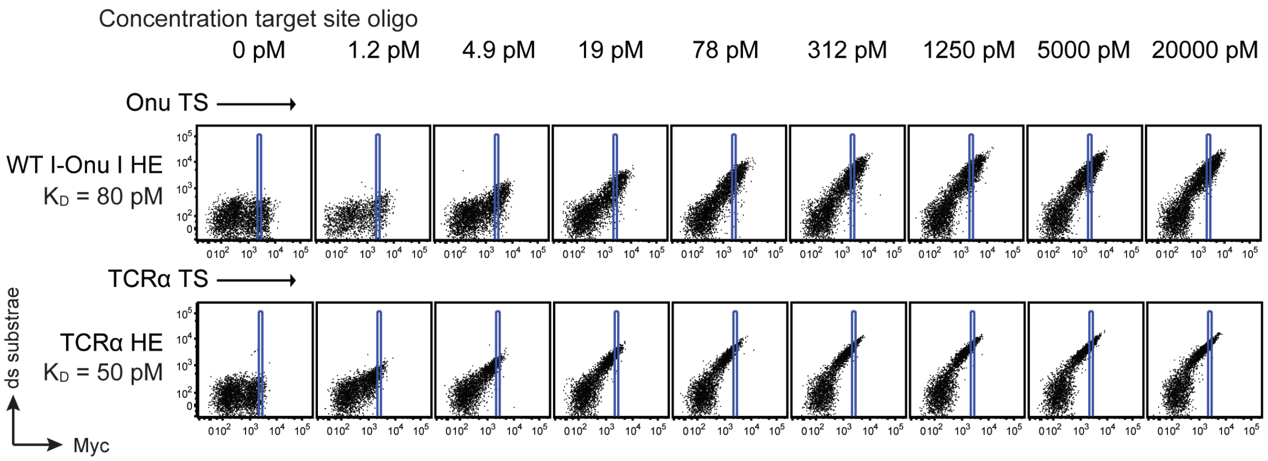
Concentration target site oligo
0 pM
1.2 pM
4.9 pM
19 pM
78 pM
312 pM
1250 pM
5000 pM
20000 pM
Onu TS
WT I-Onu I HE
KD = 80 pM
TCRα TS
TCRα HE
KD = 50 pM
ds substrae
Myc

S4b

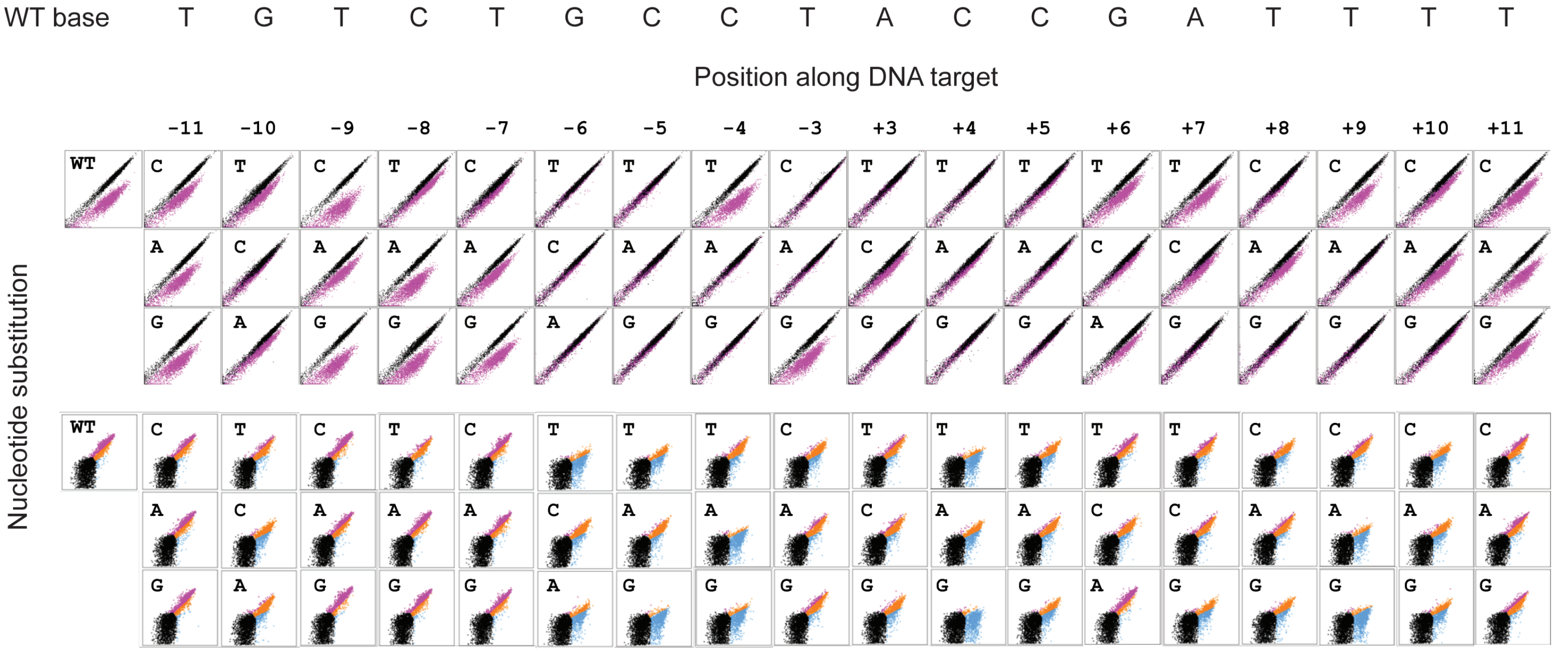
WT base
T G T C T G C C T A C C G A T T T T
Position along DNA target
-11 -10 -9 -8 -7 -6 -5 -4 -3 +3 +4 +5 +6 +7 +8 +9 +10 +11
Nucleotide substitution
WT

S4c

PWM score

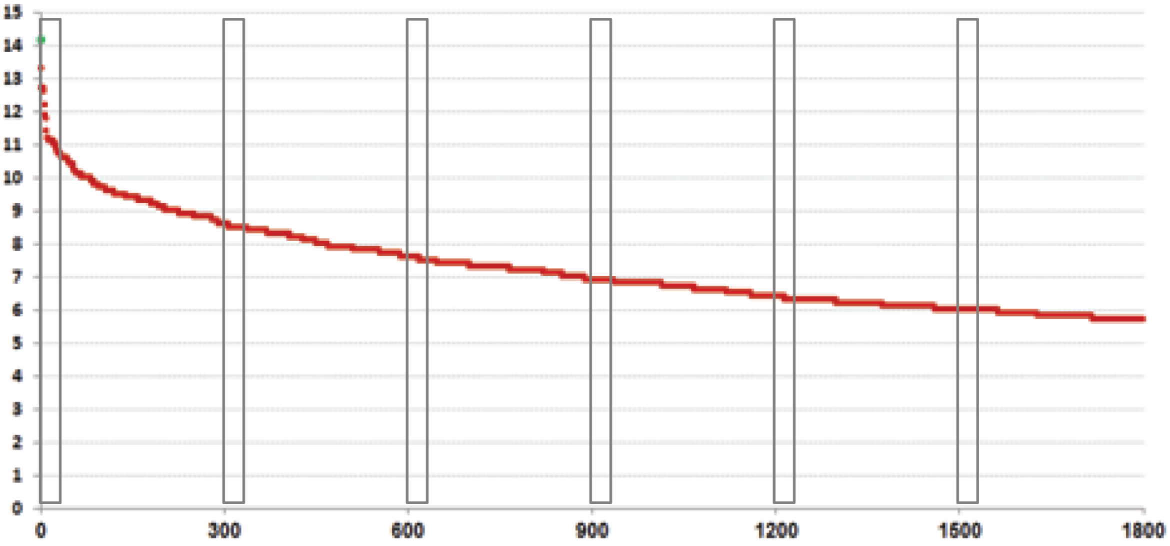

Putative genomic off-target

S4d

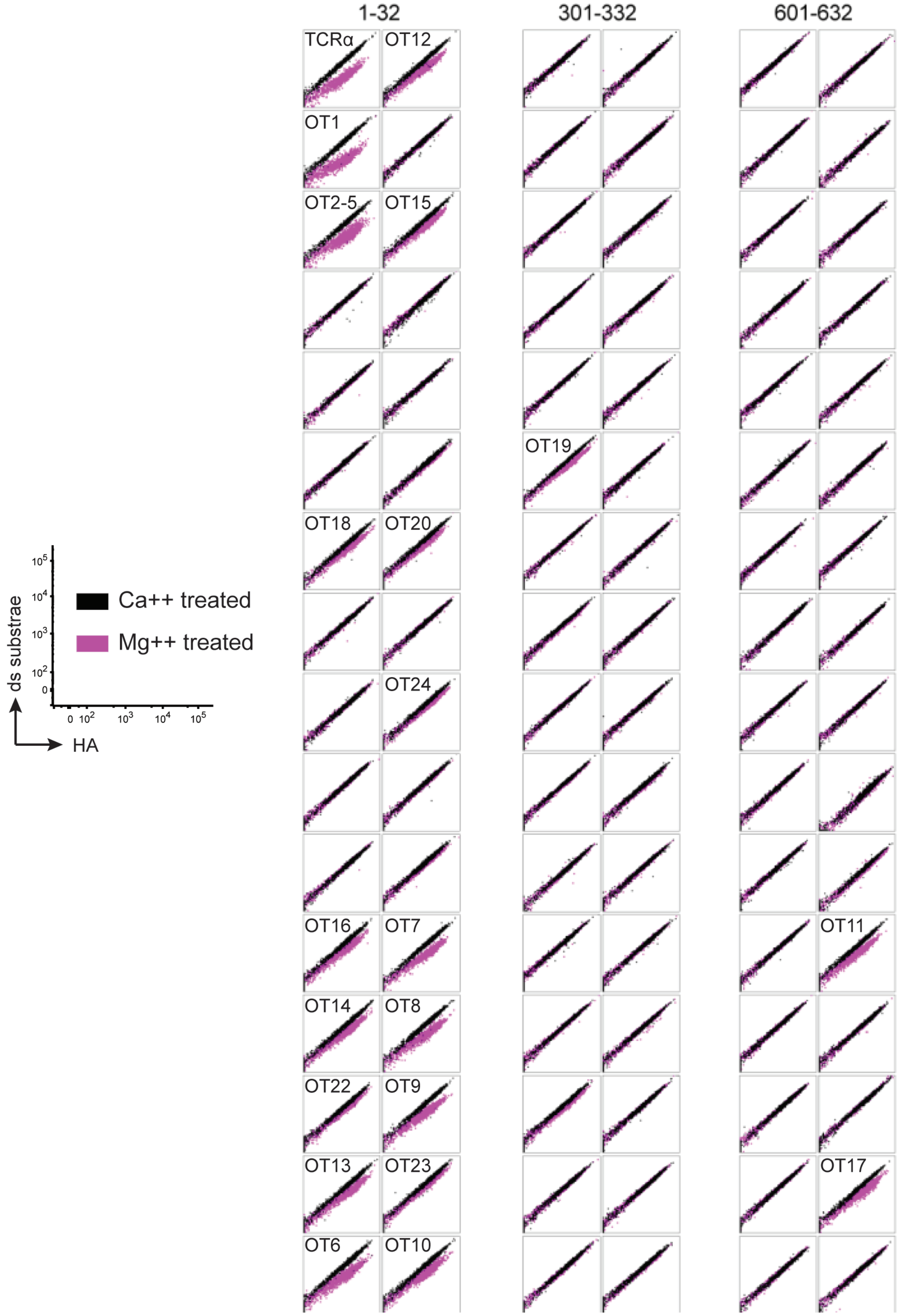
1-32
301-332
601-632
TCRα
OT12
OT1
OT2-5
OT15
OT19
OT18
OT20
OT24
OT16
OT7
OT11
OT14
OT8
OT22
OT9
OT13
OT23
OT17
OT6
OT10
ds substrae
HA
Ca++ treated
Mg++ treated

S4d, *cont.*

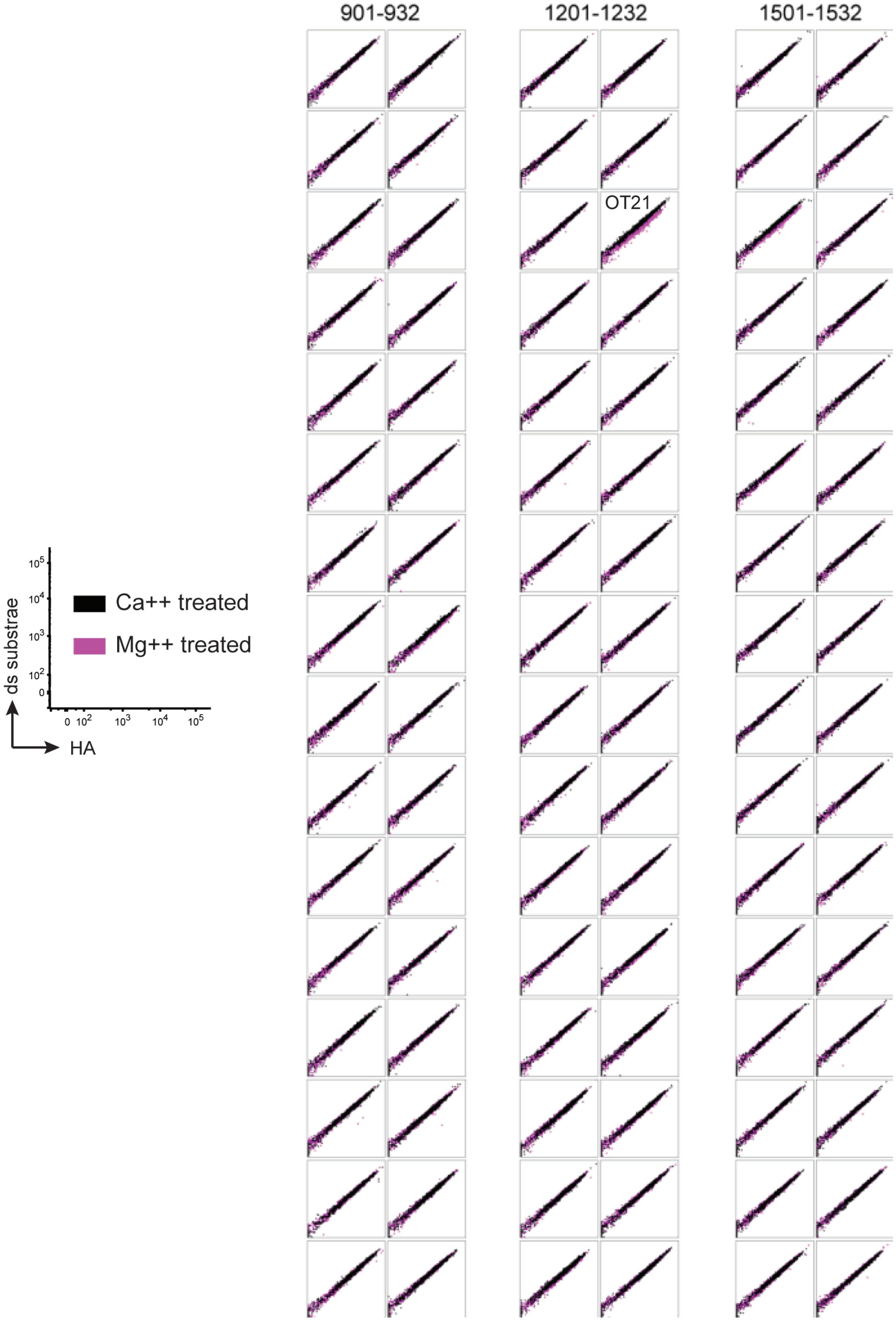

901-932
1201-1232
1501-1532
OT21
Ca++ treated
Mg++ treated
ds substrae
HA

S4e

Putative TCRα nuclease genomic off-targets

Name	Target sequence	Genomic Locus	Ca^{++}/Mg^{++}
TCRα	TGTCTGCCTATTCACCGATTTT	Ch14: (+)23016508-23016529	4.27
OT1	TGTaTGCCTtTTCACCGATTaa	Ch9: (-)21736012-2176033	5.72
OT2	TGTaTGCCTtTTCACCtATTaa	ChX: (+)112764854-112764875	3.74
OT3	TGTaTGCCTtTTCACCtATTaa	Ch20: (+)53071086-53071107	3.74
OT4	TGTaTGCCTtTTCACCtATTaa	Ch9: (+)34907420-34907441	3.74
OT5	TGTaTGCCTtTTCACCtATTaa	Ch9: (-)21119840-21119861	3.74
OT6	TGcCgGCCTtTTCACCcATcTg	Ch7: (-)112542535-112542556	2.92
OT7	GGaaTGCtTATTCACCGtTTTT	Ch14: (+)29466641-29466662	2.73
OT8	TGTgTGCtTATTCcCCtATTTa	Ch4: (-)34480172-34480193	2.68
OT9	TGgCTGCtgATTCcCCtATTTT	Ch2: (-)157897003-157897024	2.48
OT10	aGTgTGCCTATTCACCaATcTc	Ch4: (-)109209885-109209906	2.25
OT11	cacCaGCCTtTTCACCGtTcac	Ch15: (-)33161499-33161520	2.10
OT12	TGTaTGCCTtTTCcCCtATTaa	Ch6: (-)90026596-90026617	2.01
OT13	TGTaTGCCTtTTCcCCtATTaa	Ch10: (+)54726301-54726322	2.01
OT14	TGTaTGCCTtTTCcCCtATTaa	Ch20: (+)53147268-53147289	2.01
OT15	TGTaTGCCTtTTCcCCtATTaa	Ch2: (+)152020105-152020126	2.01
OT16	aGgtTGCCTtTTCACCGtTcTT	Ch1: (+)240704451-240704472	1.94
OT17	aaaaTGCCTtTTCACCtATcac	Ch13: (+)95104630-95104651	1.87
OT18	TtTaTGCCTtTTCACCGtTTTT	Ch4: (-)115725301-115725322	1.74
OT19	gtTtgGCtTATTCACCGATTTc	Ch2: (+)87036974-87036995	1.64
OT20	cGTaTGCCTtTTCcCCtATcTa	Ch5: (+)23787010-23787031	1.60
OT21	atgagGCtTATTCACCccTcaa	Ch2: (-)211575763-211575784	1.53
OT22	TGaaTGCCTtTTCACCtATTaa	Ch20: (-)15561343-15561364	1.50
OT23	gGctTGCCTtTTCACCaATTTT	ChX: (+)92468114-92468135	1.49
OT24	gaTCTGCCTtTTCACCaATTTT	Ch10: (-)109004204-109004225	1.47

Supplementary Figure 4. Characterization of the TCRα meganuclease and analysis of on-target and putative off-target cleavage

(**a**) Titration with increasing amounts (0-2000 pM final) of DNA substrates containing the I-Onu I meganuclease and TCRα meganuclease targets (directly labeled with Alexa-647 to indicate binding) incubated with yeast displaying the appropriate nuclease (labeled with anti-Myc FITC to indicate expression) indicating that the target site affinity of the engineered TCRα meganuclease is similar to the of the wild-type I-Onu I meganuclease. (**b**) Profiling cleavage (top panels) and affinity (bottom panels) specificity properties of the TCRα meganuclease, whereby each position in the DNA target sequence was serially substituted to each of the three alternative base pairs ('one-off' targets). For cleavage specificity profiling, yeast displaying the TCRα meganuclease were tethered to DNA substrates containing various one-off targets and cleavage reactions carried out in the presence of Ca^{++} (black dots - prohibits DNA cleavage) or Mg^{++} (magenta dots – facilitates hydrolysis if target site is cleavable). Cleavage of the target substrate results in a shift in Alexa-647 fluorescence by release of the non-tethered DNA fragment which contains the Alexa-647 label. For affinity specificity profiling, yeast displaying the TCRα meganuclease were incubated with a single concentration of one-off target, 20 pM, and the assay was carried out as described in S4a. The cleavage and affinity properties were quantified as described and used to generate the position-weight matrix (PWM) (20, 44). (**c**) The PWM generated in S4b was used to scan the human genome to identify putative off-target loci which, though not identical to the target sequence in the TCRα gene, contained combinations of substitutions which the one-off specificity profiling indicated could be tolerated by the meganuclease. The results of the top 1800 sites from the PWM-based scan are shown in histogram format, with the TCRα target site highlighted (green dot). (**d**) Sub-groups of putative genomic off-target sites of the TCRα meganuclease distributed along the PWM scoring range were tested for *in vitro* cleavage activity using the yeast surface display cleavage assay as described above. (**e**) Table of putative genomic off-target sites that

were further analyzed in figure S5 for *in vivo* cleavage with the TCRα meganuclease or megaTAL with the identifying name, target site sequence (lower case letters indicated base substitutions from the TCRα target), genomic position and Ca^{++}/ Mg^{++} ratio (indicating level of cleavage).

S5a

% mutNHEJ *in vivo* at TCRα and putative off-target loci (CD3- sorted populations)

Locus	Control	TCRα mn	TCRα mn + Trex2	TCRα mT	TCRα mT + Trex2
TCRα	0.225	0.062	38.609	70.599	52.219
OT1	0.352	0.259	0.343	0.378	0.349
OT2	0.659	NA	NA	0.558	0.572
OT3	0.605	0.055	0.578	0.575	0.309
OT4	0.194	0.157	0.177	0.143	0.382
OT5	0.16	0.157	4.052	0.162	0.198
OT6	0.459	0.692	0.407	0.517	0.537
OT7	0.046	NA	0.003	0.032	0.042
OT8	0.113	0.129	0.082	0.159	0.099
OT9	0.121	0.267	0.338	0.237	0.276
OT10	0.225	0.19	0.018	0.361	0.797
OT11	0.137	0.369	0.989	0.162	NA
OT12	NA	NA	NA	NA	NA
OT13	0.17	0.098	0.122	0.12	0.162
OT14	0.779	0.711	0.888	0.749	0.681
OT15	0.896	1.136	0.816	0.934	0.884
OT16	0.214	0.232	0.318	0.233	0.279
OT17	0.55	0.584	0.763	0.629	0.646
OT18	0.387	0.433	0.629	0.347	0.382
OT19	NA	NA	NA	NA	NA
OT20	0.202	0.182	0.295	0.223	0.196
OT21	0.199	0.145	0.144	0.195	0.181
OT22	0.252	0	0.213	0.257	0.232
OT23	0.352	0.383	0.302	0.464	2.078
OT24	0.132	0.121	0.154	0.135	0.148

S5b

High-throughput sequencing results for CD3- T-cells treated with TCRα nucleases

Locus	Nuclease treatment	Total read pairs (ct.)	Reads with indels (ct.)	mutNHEJ (%)
TCRα	Control	33343	75	0.225
	TCRα mn	185640	116	0.062
	TCRα mn + Trex2	37924	14642	38.609
	TCRα mT	35213	24860	70.599
	TCRα mT + Trex2	53281	27823	52.219
OT1	Control	64706	228	0.352
	TCRα mn	54885	142	0.259
	TCRα mn + Trex2	63023	216	0.343
	TCRα mT	77249	292	0.378
	TCRα mT + Trex2	55509	194	0.349
OT2	Control	39437	260	0.659
	TCRα mn	2674	0	NA
	TCRα mn + Trex2	35	0	NA
	TCRα mT	26160	146	0.558
	TCRα mT + Trex2	12592	72	0.572
OT3	Control	50284	304	0.605
	TCRα mn	54832	30	0.055
	TCRα mn + Trex2	31642	183	0.578
	TCRα mT	58227	335	0.575
	TCRα mT + Trex2	170696	528	0.309
OT4	Control	73697	143	0.194
	TCRα mn	50339	79	0.157
	TCRα mn + Trex2	63950	113	0.177
	TCRα mT	73533	105	0.143
	TCRα mT + Trex2	52156	199	0.382
OT5	Control	53083	85	0.16
	TCRα mn	30525	48	0.157
	TCRα mn + Trex2	49281	1997	4.052
	TCRα mT	50153	81	0.162
	TCRα mT + Trex2	35820	71	0.198

High-throughput sequencing results for CD3- T-cells treated with TCRα nucleases, *cont.*

Locus	Nuclease treatment	Total read pairs (ct.)	Reads with indels (ct.)	mutNHEJ (%)
OT6	Control	50281	231	0.459
	TCRα mn	30472	211	0.692
	TCRα mn + Trex2	48682	198	0.407
	TCRα mT	45648	236	0.517
	TCRα mT + Trex2	32398	174	0.537
OT7	Control	152867	70	0.046
	TCRα mn	3601	0	NA
	TCRα mn + Trex2	261288	7	0.003
	TCRα mT	176634	56	0.032
	TCRα mT + Trex2	126444	53	0.042
OT8	Control	37060	42	0.113
	TCRα mn	30336	39	0.129
	TCRα mn + Trex2	40380	33	0.082
	TCRα mT	55365	88	0.159
	TCRα mT + Trex2	39235	39	0.099
OT9	Control	72431	88	0.121
	TCRα mn	33284	89	0.267
	TCRα mn + Trex2	42061	142	0.338
	TCRα mT	48176	114	0.237
	TCRα mT + Trex2	34390	95	0.276
0T10	Control	58656	132	0.225
	TCRα mn	34713	66	0.19
	TCRα mn + Trex2	61069	11	0.018
	TCRα mT	82021	296	0.361
	TCRα mT + Trex2	62753	500	0.797
OT11	Control	69335	95	0.137
	TCRα mn	88017	325	0.369
	TCRα mn + Trex2	132750	1313	0.989
	TCRα mT	69582	113	0.162
	TCRα mT + Trex2	271	0	NA

High-throughput sequencing results for CD3- T-cells treated with TCRα nucleases, *cont.*

Locus	Nuclease treatment	Total read pairs (ct.)	Reads with indels (ct.)	mutNHEJ (%)
OT13	Control	46538	79	0.17
	TCRα mn	34752	34	0.098
	TCRα mn + Trex2	48229	59	0.122
	TCRα mT	51651	62	0.12
	TCRα mT + Trex2	38888	63	0.162
OT14	Control	56003	436	0.779
	TCRα mn	4359	31	0.711
	TCRα mn + Trex2	17337	154	0.888
	TCRα mT	58065	435	0.749
	TCRα mT + Trex2	40499	276	0.681
OT15	Control	51102	458	0.896
	TCRα mn	34060	387	1.136
	TCRα mn + Trex2	49264	402	0.816
	TCRα mT	48832	456	0.934
	TCRα mT + Trex2	42188	373	0.884
OT16	Control	48188	103	0.214
	TCRα mn	28489	66	0.232
	TCRα mn + Trex2	44036	140	0.318
	TCRα mT	44652	104	0.233
	TCRα mT + Trex2	34082	95	0.279
OT17	Control	45671	251	0.55
	TCRα mn	21749	127	0.584
	TCRα mn + Trex2	36816	281	0.763
	TCRα mT	43426	273	0.629
	TCRα mT + Trex2	34356	222	0.646
OT18	Control	42883	166	0.387
	TCRα mn	26096	113	0.433
	TCRα mn + Trex2	38627	243	0.629
	TCRα mT	43215	150	0.347
	TCRα mT + Trex2	29809	114	0.382

High-throughput sequencing results for CD3- T-cells treated with TCRα nucleases, *cont.*

Locus	Nuclease treatment	Total read pairs (ct.)	Reads with indels (ct.)	mutNHEJ (%)
OT20	Control	49529	100	0.202
	TCRα mn	34130	62	0.182
	TCRα mn + Trex2	48059	142	0.295
	TCRα mT	54767	122	0.223
	TCRα mT + Trex2	43274	85	0.196
OT21	Control	67169	134	0.199
	TCRα mn	37217	54	0.145
	TCRα mn + Trex2	54043	78	0.144
	TCRα mT	54327	106	0.195
	TCRα mT + Trex2	38189	69	0.181
OT22	Control	52013	131	0.252
	TCRα mn	978	0	NA
	TCRα mn + Trex2	33369	71	0.213
	TCRα mT	47449	122	0.257
	TCRα mT + Trex2	32321	75	0.232
OT23	Control	38903	137	0.352
	TCRα mn	51231	196	0.383
	TCRα mn + Trex2	21885	66	0.302
	TCRα mT	31672	147	0.464
	TCRα mT + Trex2	31515	655	2.078
OT24	Control	75496	100	0.132
	TCRα mn	37039	45	0.121
	TCRα mn + Trex2	68683	106	0.154
	TCRα mT	68242	92	0.135
	TCRα mT + Trex2	58278	86	0.148

S5c

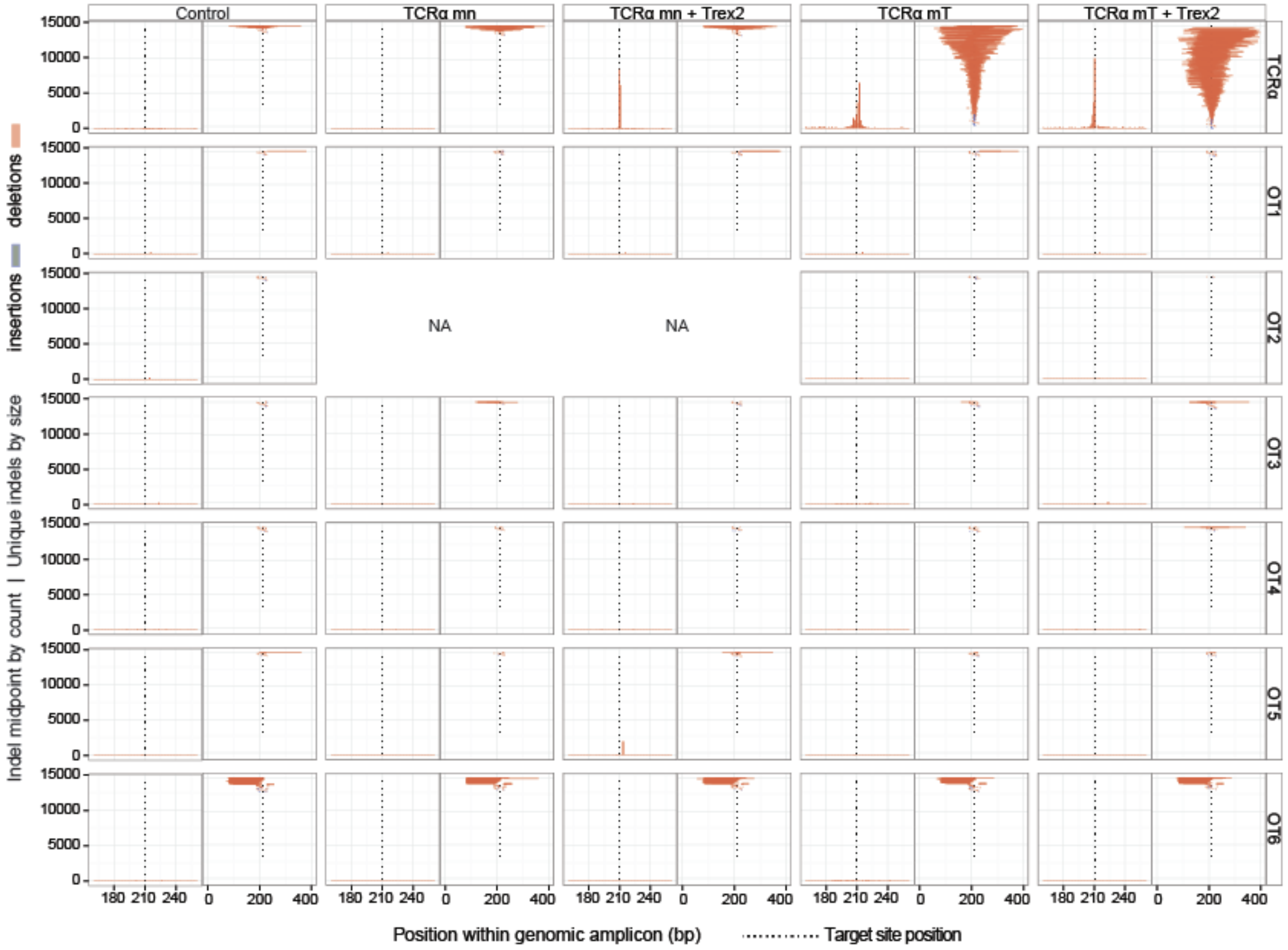

Control
TCRα mn
TCRα mn + Trex2
TCRα mT
TCRα mT + Trex2
TCRα
OT1
OT2
OT3
OT4
OT5
OT6
NA
NA
15000
10000
5000
0
180 210 240
0 200 400
deletions
insertions
Indel midpoint by count | Unique indels by size
Position within genomic amplicon (bp)
Target site position

S5c, *cont.*

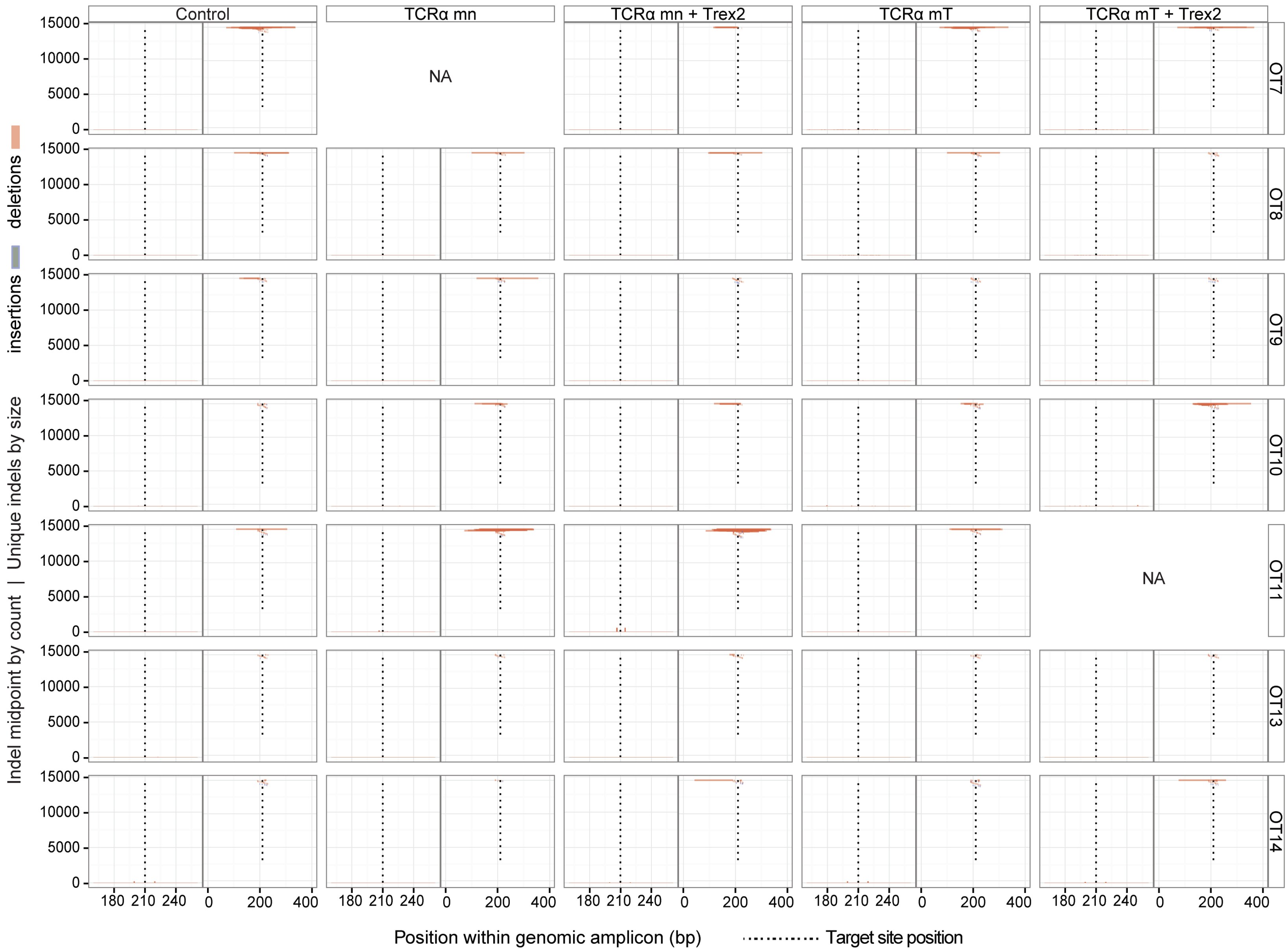

Control
TCRα mn
TCRα mn + Trex2
TCRα mT
TCRα mT + Trex2
OT7
OT8
OT9
OT10
OT11
OT13
OT14
NA
NA
15000
10000
5000
0
deletions
insertions
Indel midpoint by count | Unique indels by size
180 210 240
0 200 400
Position within genomic amplicon (bp)
Target site position

S5c, *cont.*

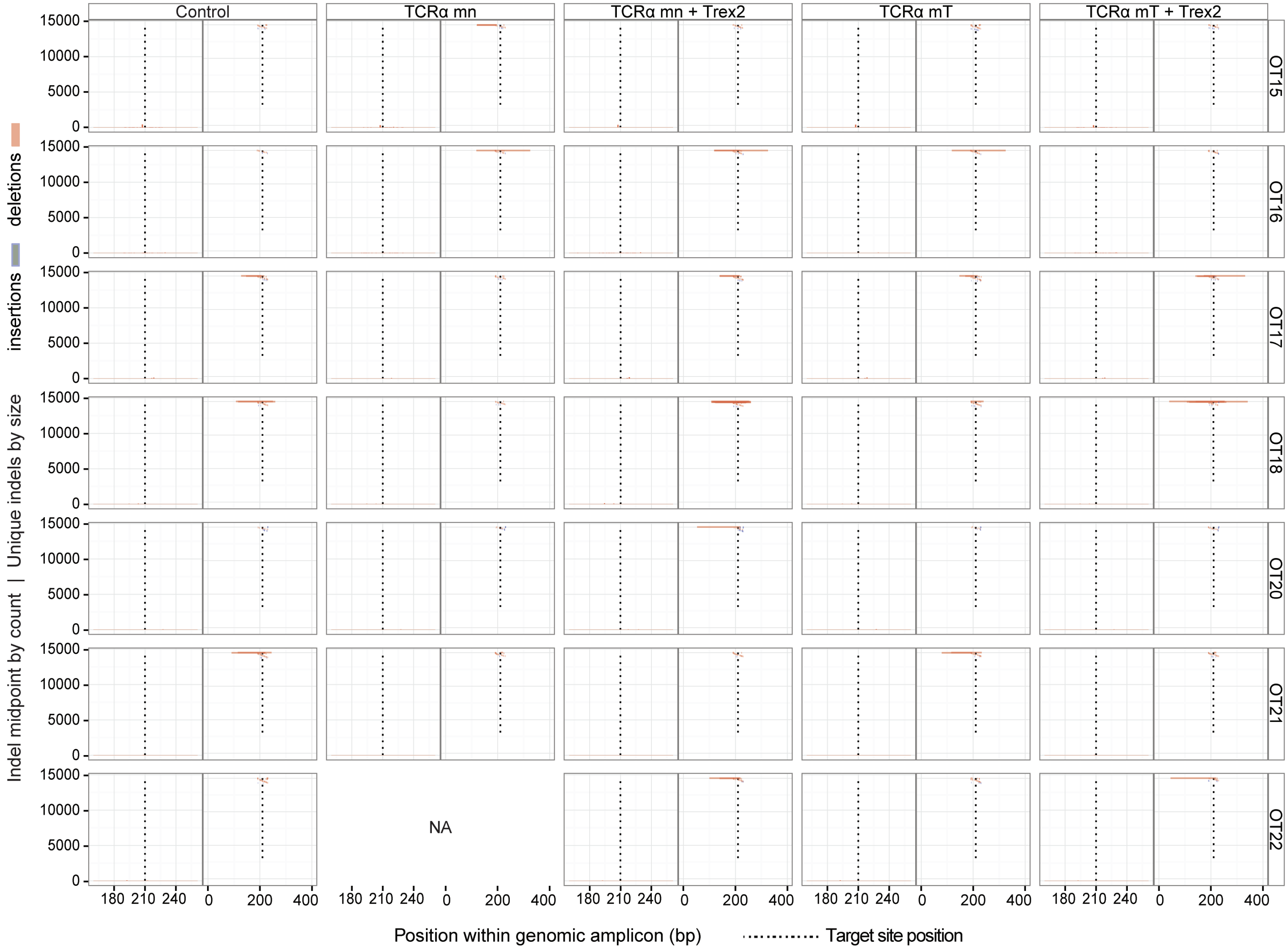
Control
TCRα mn
TCRα mn + Trex2
TCRα mT
TCRα mT + Trex2
OT15
OT16
OT17
OT18
OT20
OT21
OT22
deletions
insertions
Indel midpoint by count | Unique indels by size
15000
10000
5000
0
NA
180 210 240
0 200 400
Position within genomic amplicon (bp)
Target site position

S5c, *cont.*

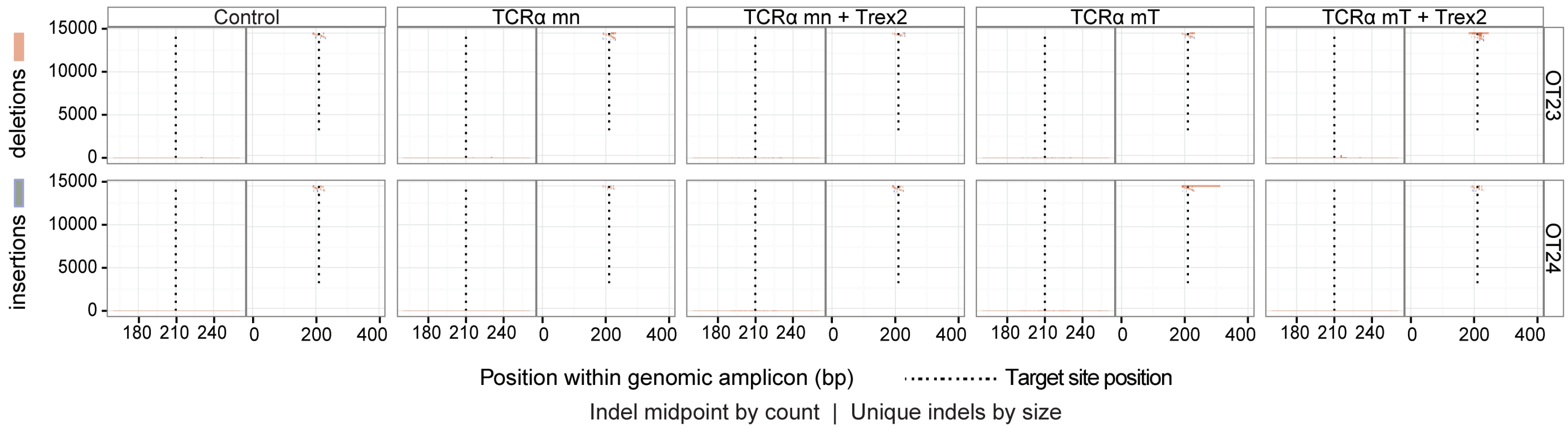

Supplementary Figure 5. High-throughput sequencing results and analysis of on-target and putative off-target cleavage in T-cells

Summary (**a**) and full statistics (**b**) of high-throughput sequencing results at TCRα and putative off-target loci from T-cells treated with the TCRα meganuclease or megaTAL +/- Trex2. (**c**) Plots showing the position and size of all unique insertions or deletions (sorted by size) found by high-throughput sequencing of megaTAL and meganuclease +/- Trex2 treated T-cells at the TCRα and putative off-target loci.

S6a

L538-Zn4-Y2 I-Ani I

VDLRTLGYSQQQQEKIKPKVRSTVAQHHEALVGHGFTHAHIVALSQHPAALGTVAVTYQHIITALP
EATHEDIVGVGKQWSGARALEALLTDAGELRGPPLQLDTGQLVKIAKRGGVTAMEAVHASRNAL
TGAPLNLTPDQVVAIAS**NG**GGKQALETVQRLLPVLCQDHGLTPDQVVAIAS**HD**GGKQALETVQR
LLPVLCQDHGLTPDQVVAIAS**NI**GGKQALETVQRLLPVLCQDHGLTPDQVVAIAS**NG**GGKQALET
VQRLLPVLCQDHGLTPDQVVAIAS**NG**GGKQALETVQRLLPVLCQDHGLTPDQVVAIAS**NI**GGKQA
LETVQRLLPVLCQDHGLTPDQVVAIAS**HD**GGKQALETVQRLLPVLCQDHGLTPDQVVAIAS**NI**GG
KQALETVQRLLPVLCQDHGLTPDQVVAIAS**HD**GGKQALETVQRLLPVLCQDHGLTPDQVVAIAS**H**
DGGKQALETVQRLLPVLCQDHGLTPDQVVAIAS**NG**GGKQALETVQRLLPVLCQDHGLTPDQVVA
IAS**NN**GGKQALETVQRLLPVLCQDHGLTPDQVVAIAS**HD**GGKQALETVQRLLPVLCQDHGLTPD
QVVAIAS**NI**GGKQALETVQRLLPVLCQDHGLTPDQVVAIAS**NN**GGKQALETVQRLLPVLCQDHGL
TPDQVVAIAS**HD**GGKQALETVQRLLPVLCQDHGLTPDQVVAIAS**NG**GGKQALESIVAQLSRPDPA
LAALTNDHLVALACLGGRPAMDAVKKGLPHAPELIRRVNRRIGERTSHRVAISRVGGSDLTYAYL
VGLYEGDGYFSITKKGKYLTYELGIELSIKDVQLIYKIKKILGIGIVSFRKRNEIEMVALRIRDKNHLKS
KILPIFEKYPMFSNKQYDYLRFRNALLSGIIYLEDLPDYTRSDEPLNSIESIINTSYFSAWLVGFIEAE
GCFSVYKLNKDDDYLIASFDIAQRDGDILISAIRKYLSFTTKVYLDKTNCSKLKVTSVRSVENIIKFLQ
NAPVKLLGNKKLQYKLWLKQLRKISRYSEKIKIPSNY

S6b

Alternative HEs

WT I-Ani I
DLTYAYLVGLFEGDGYFSITKKGKYLTYELGIELSIKDVQLIYKIKKILGIGIVSFRKRNEIEMVALRIR
DKNHLKSKILPIFEKYPMFSNKQYDYLRFRNALLSGIISLEDLPDYTRSDEPLNSIESIINTSYFSAW
LVGFIEAEGCFSVYKLNKDDDYLIASFDIAQRDGDILISAIRKYLSFTTKVYLDKTNCSKLKVTSVRS
VENIIKFLQNAPVKLLGNKKLQYKLWLKQLRKISRYSEKIKIPSNY

F13Y I-Ani I
DLTYAYLVGLYEGDGYFSITKKGKYLTYELGIELSIKDVQLIYKIKKILGIGIVSFRKRNEIEMVALRIR
DKNHLKSKILPIFEKYPMFSNKQYDYLRFRNALLSGIISLEDLPDYTRSDEPLNSIESIINTSYFSAW
LVGFIEAEGCFSVYKLNKDDDYLIASFDIAQRDGDILISAIRKYLSFTTKVYLDKTNCSKLKVTSVRS
VENIIKFLQNAPVKLLGNKKLQYKLWLKQLRKISRYSEKIKIPSNY

TCRα
SRRESINPWILTGFADAEGSFILDIRNRNNESNRYRTSLRFQITLHNKDKSILENIQSTWKVGKITN
SGDRAVMLRVTRFEDLKVIIDHFEKYPLITQKLGDYKLFKQAFSVMENKEHLKENGIKELVRIKAKM
NWGLTDELKKAFPENISKERPLINKNIPNFKWLAGFTSGDGYFGVNLKKVKGNAKVYVGLRFSIS
QHIRDKNLMNSLITYLGCGSIWEKNKSEFSWLEFVVTKFSDINDKIIPVFQENTLIGVKLEDFEDWC
KVAKLIEEKKHLTESGLDEIKKIKLNMNKGR

S6c

Alternative TAL RVDs

L538(16.5)	NG, HD, NI, NG, NG, NI, HD, NI, HD, HD, NG, NN, HD, NI, NN, HD, NG
10.5	NN, HD, NI, NN, HD, NG, NI, NG, NN, NI, NG
8.5	NN, HD, NI, NN, HD, NG, NN, NI, NG
7.5	NN, HD, NI, NN, HD, NG, NI, NG
6.5(A/A*)	NN, HD, NI, NN, HD, NG, NI
6.5(C)	NN, HD, NI, NN, HD, NG, HD
6.5(G)	NN, HD, NI, NN, HD, NG, NN
6.5(T)	NN, HD, NI, NN, HD, NG, NG
5.5	NN, HD, NI, NN, HD, NG
4.5	NN, HD, NI, NN, HD
3.5	NN, HD, NI, NN
2.5	NN, HD, NI
+9T	NN, NG, HD, NI, NG, NN, NG, NI, NI, NI, NG, NI, NI, HD, NG, NN, NG, NI
+5A+8T	NN, HD, NG, NN, NG, NG, NG, HD, NI, NG, NI, NG, NI, HD
TCRα	NI, NI, NI, NG, HD, HD, NI, NN, NG, NN, NI

Supplementary Figure 6. Coding sequences of a megaTAL, meganuclease variants and RVD array tested

(**a**) Amino acid sequence for the L538-Zn4-Y2 I-AniI megaTAL. The TAL effector and meganuclease (colored blue) sequences are separated by the Zn4 linker (underlined, VGGS). The NΔ154 and C+63 termini of the TAL effector are separated by the TALE repeat units (colored green). Alternative repeat units are underlined with dashed lines and the repeat variable diresidue (RVD) for each unit is shown in bold. (**b**) Amino acid sequences for each of the remaining homing endonucleases tested in this study: WT I-AniI, F13Y I-AniI and TCRα. (**c**) Identity of RVDs used to construct the TAL effectors used in this study.
